# Supplementary material for: Correlates of participation in community-based interventions: Evidence from a parenting program in rural China
Source: PLoS One. 2020 Sep 8;15(9):e0238841. doi: 10.1371/journal.pone.0238841 (PMC7478867; doi:10.1371/journal.pone.0238841)
Supplement: S1 File — (DOCX) [file pone.0238841.s001.docx]

**婴幼儿养育项目家长问卷（需要该婴幼儿饮食和营养第一负责人回答调查）**

**_____省____市（地区）______县_______乡/镇_______村_____小组（自然村），婴幼儿姓名 ；**

**婴幼儿父亲姓名： ；婴幼儿母亲姓名： ；被访者姓名:** ___________**，个人编码:** _____

**调查员姓名:_____________ 调查员编码：_______ 问卷编码：□□□□□□□，**

这里调查婴幼儿父母、祖父祖母、孩子的亲兄弟姐妹以及最近一年所有在家居住三个月以上的家庭成员.

**婴幼儿的饮食和营养的第一负责人姓名**__________**，个人编码**_______**；**

**婴幼儿的饮食和营养的第二负责人姓名**__________**，个人编码**_______**。**

| 个  人  编  码 |  |  |  |  |  |  |  |  |  |  |  |
| --- | --- | --- | --- | --- | --- | --- | --- | --- | --- | --- | --- |
|  | 性别：  1=男，2=女 | 与婴幼儿的关系：  1=父亲，2=母亲，3=祖父，  4=祖母，5=外祖父，6=外祖母，7=亲哥哥（姐姐）， 8=亲弟弟（妹妹）， 9=其他哥哥、姐姐、弟弟或妹妹，10=叔叔（姑姑）， 11=其他，**请说明** | 民族：  1=汉族，  2=回族，  3=蒙古族，  4=其他，**请说明** | 年龄（周岁） | 教育程度：  0=没上学，  1=小学，  2=初中，  3=高中或中专，  4=大专，  5=大学及以上 | 婚姻状况：  1=已婚，  2=离婚，  3=丧偶，  4=未婚 | 身体健康状况：  1=非常健康，2=健康，  3=身体一般，4=身体较差，5=身体很差，  6=去世  7=其他，**请说明** | 主要职业（工作）：  1=学生，2=务农，  3=打工，  4=自营工商业，  5=国家工作人员，  6=在家带宝宝，  7=其他，**请说明** | 工作地点：  1=本乡内，  2=本县非本乡，3=本省非本县，4=外省 | 现在是否在家住?  1=在，  2=不在 | 过去一年在家住的时间（月） |
| 101 | 1 | 1 |  |  |  |  |  |  |  |  |  |
| 102 | 2 | 2 |  |  |  |  |  |  |  |  |  |
| 103 |  |  |  |  |  |  |  |  |  |  |  |
| 104 |  |  |  |  |  |  |  |  |  |  |  |
| 105 |  |  |  |  |  |  |  |  |  |  |  |
| 106 |  |  |  |  |  |  |  |  |  |  |  |
| 107 |  |  |  |  |  |  |  |  |  |  |  |
| 108 |  |  |  |  |  |  |  |  |  |  |  |

| **一、婴幼儿基本情况** | | | | | | | | |  |
| --- | --- | --- | --- | --- | --- | --- | --- | --- | --- |
| **问题** | | | | | **选项/单位** | | | **答案** |  |
| 1. 婴幼儿营养健康负责人联系电话？ | | | | | 第一：**________________**  第二：**________________**  其他（与该婴幼儿的关系）：**______** | | | |  |
| 1. 婴幼儿出生日期？（**查找出生证明**） | | | | | _____年_____月_____日 | | | |  |
| 1. 婴幼儿性别? | | | | | 1=男， 2=女 | | |  |  |
| 1. 婴幼儿民族？ | | | | | 1=汉族， 2=蒙古族，  3=回族， 4=其他，**请说明** | | |  |  |
| 1. 该婴幼儿是妈妈的第几胎？ | | | | | 胎 | | |  |  |
| 1. 婴幼儿胎龄是多少周？**（查找出生证明）** | | | | | ____周____天 | | | |  |
| 1. 婴幼儿是否早产？ | | | | | 1=是， 2=否， 3=不知道 | | |  |  |
| 1. 婴幼儿生产方式? | | | | | 1=自然分娩（跳到8.5题），  2=剖宫（腹）产， 3=不知道 | | |  |  |
| 8.1是谁让你选择剖腹产？ | | | | | 1=医生选择， 2=自己选择，  3=家人选择 | | |  |  |
| 8.2 那么（医生／自己／家人）让你做剖腹产的理由是什么？ | | | | |  | | | |  |
| 8.3 是谁决定你在哪一天做剖腹产？ | | | | | 1=医生， 2=自己， 3=家人 | | |  |  |
| 8.4 你是否给该婴幼儿选择了一个出生日期？ | | | | | 1=是 2=否 | | |  |  |
| 8.5你会不会因为考虑孩子上学的问题，把孩子的生产日期提前到9月1日以前？（9月1日是开学日） | | | | | 1=会 2=不会 | | |  |  |
| 8.6 生该婴幼儿实际花了多少钱（不算报销的）？ | | | | | 元 | | |  |  |
| 8.7 国家政策为你生这个孩子总共报销了多少？ | | | | | 元 | | |  |  |
| 1. 婴幼儿出生地点？ | | | | | 1=县市级医院， 2=乡镇医院，3=计生服务机构，4=家庭，  5=其他，**请说明**，6=不知道 | | |  |  |
| 1. 婴幼儿出生时的身高？**（查找出生证明）** | | | | | 公分 | | |  |  |
| 1. 婴幼儿出生时的体重？**（查找出生证明）** | | | | | 克 | | |  |  |
| **二、婴幼儿喂养情况** | | | | | | | | |  |
| **问题** | | | | | **选项/单位** | | | **答案** |  |
| 12.该婴幼儿出生后多长时间，第一次喝母乳（早开奶情况，本表中母乳指妈妈或其他人的乳汁）？ | | | | | 1=一个小时内，2=一个小时后但一天之内，3=一周内，  4=没有喝过母乳，5=一周之后 | | |  |  |
| 12.2 该婴幼儿昨天是否吃过口服维生素补充剂或口服药剂? | | | | | | 1=是， 2=否 | | |  |
| 12.3 该婴幼儿昨天是否吃过其他口服补液? | | | | | | 1=是， 2=否 | | |  |
| 12.0该婴幼儿是否吃过母乳？ | | | | | 1=是， 2=否**（跳到15.1）** | | |  |  |
| 13.**昨天**是否给该婴幼儿吃了母乳？ | | | | | | 1=是， 2=否**（跳到14题）** | | |  |
| **问题** | | | | | **选项/单位** | | | **答案** |  |
| 13.1 **昨天**给该婴幼儿吃了几次母乳？ | | | | | 次 | | |  |  |
| 14.该婴幼儿现在是否**完全母乳**喂养（指只喝母乳，不吃别的任何食物，包括水）？ | | | | | 1=是**（完成15题跳到21.22题）**， 2=否 | | |  |  |
| 15.该婴幼儿完全母乳喂养持续时间？ | | | | | 月 | | |  |  |
| 15.1 该婴幼儿**昨天**是否喝了开水、果汁或者汤（饺子汤和米汤等）？ | | | | | 1=是， 2=否 | | |  |  |
| 16.4 该婴幼儿昨天是否喝了酸奶？ | | | | | 1=是， 2=否**（跳到16.7题）** | | |  |  |
| 16.5 该婴幼儿昨天喝了多少次酸奶？ | | | | | 次 | | |  |  |
| 16.7 该婴幼儿昨天是否喝了其它任何饮料（含豆浆、肉汤）？注意：喝过肉汤算作吃过肉，也算喝过其它任何饮料 | | | | | 1=是， 2=否 | | |  |  |
| 16.9 该婴幼儿昨天吃了多少次非配方类奶粉、袋装奶、瓶装奶或鲜奶等？ | | | | | 次 | | |  |  |
| 18.该婴幼儿是否曾经吃过配方奶粉？ | | | | | 1=是，2=否**（跳到21.1题）** | | |  |  |
| 1. 1该婴幼儿吃配方奶粉持续时间？ | | | | | 月 | | |  |  |
| 18.3该婴幼儿昨天吃了多少次配方奶粉？ | | | | | 次 | | |  |  |
| 21.1该婴幼儿昨天是否吃了米汤、粥、面汤、馒头或米饭等主食类食物？ | | | | | 1=是， 2=否 | | |  |  |
| 21.2 该婴幼儿昨天是否吃了南瓜、胡萝卜、红心红薯等里面是黄色或橙色的食物？ | | | | | 1=是， 2=否 | | |  |  |
| 21.3 该婴幼儿昨天是否吃了土豆、山药、白萝卜、白心红薯等根茎类食物？ | | | | | 1=是， 2=否 | | |  |  |
| 21.4 该婴幼儿昨天是否吃了深绿色且有叶子的蔬菜？ | | | | | 1=是， 2=否 | | |  |  |
| 21.5 该婴幼儿昨天是否吃了柿子、杏、西瓜、哈密瓜、西红柿等颜色为红黄色的水果？ | | | | | 1=是， 2=否 | | |  |  |
| 21.6 该婴幼儿昨天是否吃了其它的水果或蔬菜？ | | | | | 1=是， 2=否 | | |  |  |
| 21.7 该婴幼儿昨天是否吃了肝、肾、心等内脏类食物？ | | | | | 1=是， 2=否 | | |  |  |
| 21.8 该婴幼儿昨天是否吃了肉或肉制品（如鸡、鸭、猪、牛、羊等肉）？ | | | | | 1=是， 2=否 | | |  |  |
| 21.9该婴幼儿昨天是否吃了鸡蛋？ | | | | | 1=是， 2=否 | | |  |  |
| 21.10 该婴幼儿昨天是否吃了鱼类、贝类或海鲜类食物？ | | | | | 1=是， 2=否 | | |  |  |
| 21.11 该婴幼儿昨天是否吃了扁豆、豇豆等豆类、豆制品或坚果类食物？ | | | | | 1=是， 2=否 | | |  |  |
| 21.12 该婴幼儿昨天是否吃了奶酪（片）、酸奶或其他奶制品类食物？ | | | | | 1=是， 2=否 | | |  |  |
| 21.13该婴幼儿昨天是否吃了油（包括炒菜用的油）、肥肉等脂肪类的食物？ | | | | | 1=是， 2=否 | | |  |  |
| **问题** | | | | | **选项/单位** | | | **答案** |  |
| 21.14该婴幼儿昨天是否吃了饼干、点心、糖、巧克力、蛋糕等食物？ | | | | | 1=是， 2=否 | | |  |  |
| 21.15该婴幼儿昨天是否吃了添加了调料（如辣椒、香菜、姜、蒜、鱼粉、虾皮等）的食物？ | | | | | 1=是， 2=否 | | |  |  |
| 21.16该婴幼儿昨天是否吃了任何固体（如米饭、包子等）、半固体（稠的，如粥等），或软的(如面糊、果泥、菜泥等）食物？ | | | | | 1=是， 2=否 | | |  |  |
| 21.17该婴幼儿昨天吃了几次固体（如米饭、包子等）、半固体（稠的，如粥等）或软的（如面糊、果泥、菜泥等）食物？ | | | | | 次 | | |  |  |
| 21.22 该婴幼儿是否拿到了国家免费发的营养包？ | | | | | 1=是； 2=否**（跳到31题）** | | |  |  |
| 21.23 该婴幼儿多大的时候开始拿到国家免费发的营养包？ | | | | | 月 | | |  |  |
| 21.24 截至目前，你给该婴幼儿共拿了多少袋国家免费发的营养包？ | | | | | 袋 | | |  |  |
| 21.25 过去一个星期，您给该婴幼儿喂了多少袋国家免费发的营养包？ | | | | | 袋 | | |  |  |
| 21.26 截至目前，您给该婴幼儿喂了多少袋国家免费发的营养包？ | | | | | 袋 | | |  |  |
| 31.在**最近一个月**，您给该宝宝买维生素及微量元素的补充品共花了多少钱？ | | | | | 元 | | |  |  |
| 32. 在**最近一个月**，您给该宝宝买配方奶粉共花了多少钱？ | | | | | 元 | | |  |  |
| **三、婴幼儿妇幼保健和卫生情况** | | | | | | | | |  |
| **问题** | | **选项/单位** | | | | | | **答案** |  |
| 45. 婴幼儿是否参加过免费体检？ | | 1=是， 2=否， 3=不知道 | | | | | |  |  |
| 45.1 婴幼儿有预防接种卡吗？ | | 1=有， 2=没有**（跳到48题）**，  3=不知道 | | | | | |  |  |
| 45.2 婴幼儿是否按时打疫苗？**（查预防接种卡）** | | 1=是， 2=否 | | | | | |  |  |
| 48.婴幼儿过去体检时是否发现过什么严重疾病？ | | 0=没做过体检，  1=是， 2=否，  3=不知道 | | | | | |  |  |
| 49.如果发现，是什么疾病？（**请说明，如果没有发现疾病，请填“无”**） | |  | | | | | | |  |
| **四、婴幼儿身体健康情况** | | | | | | | | |  |
| **问题** | | **选项/单位** | | | | | | **答案** |  |
| 1. 在**最近两个星期**，该婴幼儿有没有发烧？ | | 1=有， 2=没有 | | | | | |  |  |
| 1. 在**最近两个星期**，该婴幼儿有没有咳嗽？ | | 1=有， 2=没有 | | | | | |  |  |
| **问题** | | | **选项/单位** | | | | | **答案** |  |
| 1. 在**最近两个星期**，该婴幼儿有没有拉肚子？ | | 1=有， 2=没有 | | | | | |  |  |
| 1. 在**最近两个星期**，该婴幼儿有没有因为积食而不舒服？ | | | 1=有， 2=没有 | | | | |  |  |
| 1. 在**最近两个星期**，该婴幼儿有没有上呼吸道感染（感冒）？ | | | 1=有， 2=没有 | | | | |  |  |
| 1. 在**最近两个星期**，该婴幼儿共生过几次病？ | | | 次 | | | | |  |  |
| 59.1 在**最近两个星期**，该婴幼儿生了几天病？ | | | 天 | | | | |  |  |
| 1. 在**最近两个星期**，该婴幼儿看病共花了多少钱？ | | | 元 | | | | |  |  |
| 60.1 在**最近两个星期**，该婴幼儿得的最严重的一次病是什么病？ | | | 说明 | | | | |  |  |
| 60.2 在**最近两个星期**，该婴幼儿看最严重的一次病花了多少钱？ | | | 元 | | | | |  |  |
| 60.3 **调查时**该宝宝是否身体不舒服或生病？ | | | 1=是， 2=否 | | | | |  |  |
| 61 你对县计生干部的看法？ | | | 1=非常喜欢， 2=喜欢，  3=一般， 4=讨厌，  5=非常讨厌 6=没接触过 | | | | |  |  |
| 62 你对村计生干部的看法？ | | | 1=非常喜欢， 2=喜欢，  3=一般， 4=讨厌，  5=非常讨厌 6=没接触过 | | | | |  |  |
| **五、婴幼儿父母（主要看护人）其它养育行为和计划** | | | | | | | | |  |
| **问题** | | | **选项** | | | | | **答案** |  |
| 74.1你们家宝宝经常会跟几个6-18月龄的宝宝一起玩？ | | | 个 | | | | |  |  |
| 74.2你们家宝宝经常会跟几个18-30月龄的宝宝一起玩？ | | | 个 | | | | |  |  |
| 74.3你们家宝宝经常会跟几个30-42月龄的宝宝一起玩？ | | | 个 | | | | |  |  |
| 1. 你昨天跟其他宝宝的家长在一起呆了多长时间？ | | | 小时 | | | | |  |  |
| 81.1 昨天你用玩具和宝宝一起玩游戏了吗？ | | | 1=玩过， 2=没玩过 | | | | |  |  |
| 81.2 昨天你给宝宝讲过故事吗？ | | | 1=讲过， 2=没讲过 | | | | |  |  |
| 81.3 昨天给宝宝讲故事时，用故事书了吗？ | | | 1=用了， 2=没用 | | | | |  |  |
| 81.4 昨天你教宝宝唱过儿歌吗？ | | | 1=唱过， 2=没唱过 | | | | |  |  |
| 82.1 宝宝出生到现在，妈妈一共**全职在家**带了宝宝几个月？ | | | 月 | | | | |  |  |
| **问题** | | | **选项/单位** | | | | | **答案** |  |
| 82.2宝宝出生到现在，妈妈**一边工作一边在家**带宝宝几个月？ | | | 月 | | | | |  |  |
| 82.3 妈妈一边工作一边在家带宝宝时每月收入多少？（没有这种情况填“0”） | | | 元 | | | | |  |  |
| 82.4 宝宝出生到现在，妈妈**全职外出**工作（不住家里）几个月？ | | | 月 | | | | |  |  |
| 82.5妈妈全职外出工作（不住家里）每月收入多少？（没有这种情况填“0”） | | | 元 | | | | |  |  |
| 82.6宝宝几个月大时，妈妈第一次全职外出工作（不住在家里或不带宝宝）？（没有外出过画“\”） | | | 月 | | | | |  |  |
| 84.婴幼儿母亲现在在家带宝宝吗？ | | | 1=是， 2=否 | | | | |  |  |
| 85.1 宝宝出生到现在，爸爸一共全职在家带了宝宝几个月？ | | | 月 | | | | |  |  |
| 85.2宝宝出生到现在，爸爸一边工作一边在家带宝宝几个月？ | | | 月 | | | | |  |  |
| 85.3 爸爸一边工作一边在家带宝宝时每月收入多少？（没有这种情况填“0”） | | | 元 | | | | |  |  |
| 85.4 宝宝出生到现在，爸爸全职外出工作（不住家里）几个月？ | | | 月 | | | | |  |  |
| 85.5爸爸全职外出工作（不住家里）每月收入多少？（没有这种情况填“0”） | | | 元 | | | | |  |  |
| 85.6宝宝几个月大时，爸爸第一次全职外出工作（不住在家里或不带宝宝）？（没有外出过画“\”） | | | 月 | | | | |  |  |
| 85.7爸爸现在在家带宝宝吗？ | | | 1=是， 2=否 | | | | |  |  |
| 85.8家里现在有几个劳动力在家务农或在家做自营工商业？ | | | 个 | | | | |  |  |
| 85.9今年一年，家里农业生产经营收入大约有多少？ | | | 元 | | | | |  |  |
| 85.10 今年一年，家里非农自营工商业收入大约有多少？ | | | 元 | | | | |  |  |
| **六、婴幼儿父母（主要看护人）养育信息来源情况** | | | | | | | | |  |
| **问题** | | | | | **选项/单位** | | | **答案** |  |
| 1. 你从哪些渠道获取儿童养育方面的信息? **(可多选)** | | | | | 1=家人； 2=朋友；3=村医；  4=计生专干；5=妇女主任  6=其他养育专家； 7=书籍；  8=电视； 9=网络；  10=其他；请说明 | | |  |  |
| 1. 你过去是否参加过儿童养育知识方面培训？ | | | | | 1=是， 2=否， 3=不知道 | | |  |  |
| 1. 过去一年里，是否有人和你说过如何教宝宝远离危险物品或环境（如农药、水塘、火、插座等）？ | | | | | | | 1=是，  2=否， 3=不知道 |  |  |
| 1. 过去一年里，是否有人和你说过宝宝在生活自理（如洗漱和穿衣）方面力所能及并自己可以做的事情？ | | | | | | 1=是，  2=否， 3=不知道 | |  |  |
| 1. 过去一年里，是否有人和你说过宝宝所能理解和使用词语的情况？ | | | | | | 1=是，  2=否， 3=不知道 | |  |  |
| 1. 过去一年里，是否有人和你说过给宝宝读故事书的重要性？ | | | | | | 1=是，  2=否， 3=不知道 | |  |  |
| 1. 过去一年里，是否有人和你说过读故事书给宝宝听的事情？ | | | | | | 1=是，  2=否， 3=不知道 | |  |  |
| 1. 过去一年里，是否有人和你说过和宝宝唱儿歌的事情？ | | | | | | 1=是，  2=否， 3=不知道 | |  |  |
| 1. 过去一年里，是否有人和你说过和宝宝一起玩游戏的事情？ | | | | | | 1=是，  2=否， 3=不知道 | |  |  |
| 1. 过去一年里，是否有人和你说过宝宝是如何学习与其他宝宝相处的？ | | | | | | 1=是，  2=否， 3=不知道 | |  |  |
| 1. 过去一年里，是否有人和你说过如何帮助宝宝理解规矩和守规矩的一些方法（如限定时间做某件事）？ | | | | | | 1=是，  2=否， 3=不知道 | |  |  |
| **七、婴幼儿父母（主要看护人）养育环境情况** | | | | | | | | |  |
| **问题** | | | | **选项/单位** | | | | **答案** |  |
| 1. 你家宝宝有独立的儿童活动区（如铺软垫的区域）吗？ | | | | 1=一直都有，2=经常会有，  3=有时候有，4=很少才有，5=没有 | | | |  |  |
| 1. 您家屋里有手机信号吗？ | | | | 1=有，2=没有 | | | |  |  |
| 1. 您家有人抽烟吗？ | | | | 1=有，2=没有（**跳到102.1题**） | | | |  |  |
| 1. 如果有人抽烟，在家里（室内）抽烟的情况？ | | | | 1=从不，2=有时，3=经常 | | | |  |  |
| 102.1 宝宝父亲有几个亲兄弟姐妹？ | | | | 个 | | | |  |  |
| 102.2 宝宝父亲的亲兄弟姐妹有几个住在本村？ | | | | 个 | | | |  |  |
| 102.3 宝宝父亲的亲兄弟姐妹有几个在外打工？ | | | | 个 | | | |  |  |
| 102.4 宝宝父亲的亲兄弟姐妹共有几个孩子？ | | | | 个 | | | |  |  |
| 102.5 其中几个孩子和该宝宝住在同村？ | | | | 个 | | | |  |  |
| 102.6 其中几个和宝宝住在同村的孩子小于3岁？ | | | | 个 | | | |  |  |
| 102.7 宝宝母亲有几个亲兄弟姐妹？ | | | | 个 | | | |  |  |
| 102.8 宝宝母亲的亲兄弟姐妹有几个住在本村？ | | | | 个 | | | |  |  |
| 102.9 宝宝母亲的亲兄弟姐妹有几个在外打工？ | | | | 个 | | | |  |  |
| 102.10 宝宝母亲的亲兄弟姐妹共有几个孩子？ | | | | 个 | | | |  |  |
| 102.11其中几个孩子和该宝宝住在同村？ | | | | 个 | | | |  |  |
| **问题** | | | | **选项** | | | | **答案** |  |
| 102.12其中几个和宝宝住在同村的孩子小于3岁？ | | | | 个 | | | |  |  |
| 102.13 家庭成员之间一直合得来吗？ | | | | 1=是，2=否 | | | |  |  |
| 102.14 家庭成员总是衷心的相互支持吗？ | | | | 1=是，2=否 | | | |  |  |
| 102.15 家庭成员会经常互相责备和指责吗？ | | | | 1=经常会，2=有时会，3=不会 | | | |  |  |
| 102.16 家庭成员有矛盾时，会大声争吵吗？ | | | | 1=经常会，2=有时会，3=不会 | | | |  |  |
| 102.17 有时家庭成员发怒时会摔东西吗？ | | | | 1=经常会，2=有时会，3=不会 | | | |  |  |
| 102.18 有时家庭成员发怒时会打人吗？ | | | | 1=经常会，2=有时会，3=不会 | | | |  |  |
| **八、婴幼儿父母（主要看护人）养育行为情况** | | | | | | | | |  |
| **问题** | | | | | **选项** | | | **答案** |  |
| 111.**过去两天**你有多少次通过拥抱、亲吻或其他方式向你家宝宝表达亲密的感情？ | | | | | 次 | | |  |  |
| 112.一般每天你家宝宝看电视或视频（包括手机游戏）的时间。 | | | | | 分钟（没有选0） | | |  |  |
| 113.一般每天你家宝宝自己独自玩的时间。 | | | | | 分钟（没有选0） | | |  |  |
| 113.2**昨天**你单独陪宝宝玩游戏的时间。 | | | | | 分钟（没有写0） | | |  |  |
| 113.3**昨天**你（或其他成人）单独和宝宝讲故事（说话）的时间。 | | | | | 分钟（没有写0） | | |  |  |
| 113.4**昨天**你（或其他成人）单独和宝宝读故事书的时间。 | | | | | 分钟（没有写0） | | |  |  |
| 113.5**昨天**你（或其他成人）单独和宝宝唱儿歌的时间。 | | | | | 分钟（没有写0） | | |  |  |
| 113.6**昨天**你家宝宝哭过几次？ | | | | | 次 | | |  |  |
| 113.7**昨天**宝宝尿湿裤子的次数？ | | | | | 次 | | |  |  |
| 113.8**昨天**宝宝大便弄脏衣物的次数？ | | | | | 次 | | |  |  |
| 113.9**昨天**你带宝宝时生过几次气？ | | | | | 次 | | |  |  |
| 113.10**昨天**你（或其他成人）大声说（吼或骂）过几次宝宝？ | | | | | 次 | | |  |  |
| 113.11**昨天**你（或其他成人）打过几次宝宝？ | | | | | 次 | | |  |  |
| 113.12**昨天**你家宝宝摔了几次？ | | | | | 次 | | |  |  |
| 113.13**昨天**你家宝宝和别的宝宝有过几次矛盾（有一个宝宝哭了）？ | | | | | 次 | | |  |  |
| 113.14 你家宝宝会说脏话（只要说脏字就算）吗？ | | | | | 1=会，2=不会 | | |  |  |
| 113.15你家宝宝会朝人吐口水吗（只要有动作就算）？ | | | | | 1=会，2=不会 | | |  |  |
| 113.16你家宝宝会动手打人（抓人、咬人或挠人等）吗（只要有动作就算）？ | | | | | 1=会，2=不会 | | |  |  |
| **问题** | | | | | **选项** | | | **答案** |  |
| 113.17你家宝宝会拿东西打人（棍子、石子等）吗（只要有动作就算）？ | | | | | 1=会，2=不会 | | |  |  |
| 113.18**过去一年**给宝宝买玩具花了多少钱？ | | | | | 元 | | |  |  |
| 113.19**过去一年**给宝宝买书花了多少钱？ | | | | | 元 | | |  |  |
| 114.在管教宝宝时，你使用提高声调或吼的方式的情况。 | | | | | 1=经常，2=有时，3=很少，  4=从不，5=不知道 | | |  |  |
| 115.在管教宝宝时，你使用打宝宝屁股的方式的情况。 | | | | | 1=经常，2=有时，3=很少，  4=从不，5=不知道 | | |  |  |
| 116.在管教宝宝时，你使用拿走宝宝的玩具或宝宝其他想要的东西的方式的情况。 | | | | | 1=经常，2=有时，3=很少，  4=从不，5=不知道 | | |  |  |
| 117.在管教宝宝时，你使用限定时间终止他正在做的事情（如看电视、玩游戏等）的方式的情况。 | | | | | 1=经常，2=有时，3=很少，  4=从不，5=不知道 | | |  |  |
| 118.在管教宝宝时，你使用向宝宝解释为什么他的行为是不适当的这一方式的情况。 | | | | | 1=经常，2=有时，3=很少，  4=从不，5=不知道 | | |  |  |
| **九、婴幼儿父母（主要看护人）对儿童养育的看法和评价** | | | | | | | | |  |
| **问题** | **选项** | | | | | | | **答案** |  |
| 1. 从上个月的情况看，你对“我真的很享受和宝宝在一起”这句话的看法。 | 1=完全不正确，2=少数正确，  3=一半一半，4=大多正确，5=完全正确 | | | | | | |  |  |
| 1. 从上个月的情况看，你对“我和我家宝宝相处的很好”这句话的看法。 | 1=完全不正确，2=少数正确，  3=一半一半，4=大多正确，5=完全正确 | | | | | | |  |  |
| 1. 从上个月的情况看，你对“和宝宝在一起时我感到很烦”这句话的看法。 | 1=完全不正确，2=少数正确，  3=一半一半，4=大多正确，5=完全正确 | | | | | | |  |  |
| 1. 从上个月的情况看，你对“和宝宝在一起的时候我感到紧张（压力大）”这句话的看法。 | 1=完全不正确，2=少数正确，  3=一半一半，4=大多正确，5=完全正确 | | | | | | |  |  |
| 1. 从上个月的情况看，你对“当我和宝宝说话时总是受到宝宝忽视”这句话的看法。 | 1=完全不正确，2=少数正确，  3=一半一半，4=大多正确，5=完全正确 | | | | | | |  |  |
| 1. 从上个月的情况看，你对“我不知道如何从儿童想问题的角度和宝宝进行沟通”这句话的看法。 | 1=完全不正确，2=少数正确，  3=一半一半，4=大多正确，5=完全正确 | | | | | | |  |  |
| 1. 从上个月的情况看，你对“和宝宝在一起做游戏非常有趣好玩”这句话的看法. | 1=完全不正确，2=少数正确，  3=一半一半，4=大多正确，5=完全正确 | | | | | | |  |  |
| 1. 你和你家宝宝在一起玩对你来说重要性如何？ | 1=完全不正确，2=少数正确，  3=一半一半，4=大多正确，5=完全正确 | | | | | | |  |  |
| 1. 你对“我知道如何和宝宝一起玩”这句话的看法。 | 1=完全不正确，2=少数正确，  3=一半一半，4=大多正确，5=完全正确 | | | | | | |  |  |
| **问题** | **选项/单位** | | | | | | | **答案** |  |
| 1. 你和你家宝宝读故事书或看故事书对你来说重要性如何？ | 1=完全不正确，2=少数正确，  3=一半一半，4=大多正确，5=完全正确 | | | | | | |  |  |
| 1. 你对“我知道如何和宝宝一起看故事书”这句话的看法。 | 1=完全不正确，2=少数正确，  3=一半一半，4=大多正确，5=完全正确 | | | | | | |  |  |
| 1. 你对“帮助宝宝认识周围的世界是父母和监护人的责任”这句话的看法。 | 1=完全不正确，2=少数正确，  3=一半一半，4=大多正确，5=完全正确 | | | | | | |  |  |
| 1. 你家宝宝将来在学校表现好对你来说重要性如何？ | 1=完全不正确，2=少数正确，  3=一半一半，4=大多正确，5=完全正确 | | | | | | |  |  |
| **十、婴幼儿家庭基本情况** | | | | | | | | |  |
| **问题** | | | | | **选项** | | | **答案** |  |
| 133.您家离村委会的距离 | | | | | 米 | | |  |  |
| 134.您家是不是享受过农村低保补贴？ | | | | | 1=是， 2=否 | | |  |  |
| 135.您家一年的收入大概有多少？ | | | | | 元 | | |  |  |
| 136.您家的房屋建筑面积有多少平方米？ | | | | | 平方米 | | |  |  |
| 137.您家的房屋现在估价值有多少万元? | | | | | 1=1万以内， 2=1-5万，  3=5-10万， 4=10-30万，  5=30万以上 | | |  |  |
| 138.您家里有自来水吗？ | | | | | 1=有， 2=没有 | | |  |  |
| 139.您家里有抽水马桶吗？ | | | | | 1=有， 2=没有 | | |  |  |
| 140.您家里有热水器吗？ | | | | | 1=有， 2=没有 | | |  |  |
| 141.您家里有洗衣机吗？ | | | | | 1=有， 2=没有 | | |  |  |
| 142.您家里有电脑吗？ | | | | | 1=有， 2=没有 | | |  |  |
| 143.您家里能够上互联网吗？ | | | | | 1=有， 2=没有 | | |  |  |
| 144.您家里有冰箱吗？ | | | | | 1=有， 2=没有 | | |  |  |
| 145.您家里有空调吗？ | | | | | 1=有， 2=没有 | | |  |  |
| 146.您家里有摩托车/电动车吗？ | | | | | 1=有， 2=没有 | | |  |  |
| 147.您家里有小轿车/货车吗？ | | | | | 1=有， 2=没有 | | |  |  |
| 148.宝宝妈妈以前有没有出去打工？ | | | | | 1=有， 2=没有（跳到153题） | | |  |  |
| 149.如果出去打过工，宝宝妈妈有没有做过保姆？ | | | | | 1=有， 2=没有 | | |  |  |
| 150.宝宝妈妈外出打工时是否了解或关注过城里人怎么带宝宝？ | | | | | 1=有， 2=没有 | | |  |  |
| 151.如果给每个月1000元的补贴，您是否愿意做养育中心的管理员 | | | | | 1=有， 2=没有 | | |  |  |
| 152.如果给每个月1500元的补贴，您是否愿意做养育中心的管理员 | | | | | 1=有， 2=没有 | | |  |  |
