# Supplementary material for: Correlates of participation in community-based interventions: Evidence from a parenting program in rural China
Source: PLoS One. 2020 Sep 8;15(9):e0238841. doi: 10.1371/journal.pone.0238841 (PMC7478867; doi:10.1371/journal.pone.0238841)
Supplement: S2 File — (DOCX) [file pone.0238841.s002.docx]

**Nourish the Future Program Caregiver Survey (to be answered by the main caregiver)**

**____Province____City____County____Township____Village___Group，Child name_______;**

**Father name_________; Mother name_______; Interviewee name________; Family member ID____**

**Interviewer name_________; Interviewer ID___________; Household ID_____________;**

**Family roster (**main members of your household and other household members living in home more than 3 months**)**

**Primary caregiver name__________, family member ID______;**

| Family mem-ber ID | **01** | **02** | **03** | **04** | **05** | **06** | **07** | **08** | **09** | **10** | **11** |
| --- | --- | --- | --- | --- | --- | --- | --- | --- | --- | --- | --- |
|  | Gender  1=Male,  2=Female; | Relationship with baby  1= Father,  2= Mother,  3= Paternal, grandfather,  4= Paternal grandmother,  5= Maternal grandfather,  6= Maternal grandmother,  7= Brother,  8= Sister,  9= Other siblings,  10= Uncle (Aunt),  11=Other, please specify | Ethnicity  1=Han,  2=Hui,  3=Mong-ols,  4=Other, please specify | Age  (year) | Highest education  0= no education,  1= Primary school,  2= Junior high school,  3=Senior high school or equivalent,  4= Associate degree,  5= Bachelor’s degree or above | Marital status  1= Married,  2=Divorced,  3= Widowed,  4= Unmarried | Overall health status  1=Very healthy,  2=Healthy,  3= Fair, 4= Poor,  5= Very poor, 6= Deceased, 7= Other, please specify | Primary occupation  1= student  2= farming  3= working for others  4= self-employed business  5= government worker  6= caregiver for children  7= other, please specify | Occupation location  1= home township, 2= home county but not home township, 3= home province but not home county,  4= Outside home province | Living at home now  1= Yes,  2= No | Months living at home last year |
| 101 |  |  |  |  |  |  |  |  |  |  |  |
| 102 |  |  |  |  |  |  |  |  |  |  |  |
| 103 |  |  |  |  |  |  |  |  |  |  |  |
| 104 |  |  |  |  |  |  |  |  |  |  |  |
| 105 |  |  |  |  |  |  |  |  |  |  |  |
| 106 |  |  |  |  |  |  |  |  |  |  |  |
| 107 |  |  |  |  |  |  |  |  |  |  |  |
| 108 |  |  |  |  |  |  |  |  |  |  |  |

**Secondary caregiver name_________, family member ID______;**

| **1． Child basic information** | | | | | |  |
| --- | --- | --- | --- | --- | --- | --- |
| **Questions** | | | **Answer Options** | | **Answer** |  |
| 1. Caregiver contact information | | | Primary：**________________**  Secondary：**________________**  Other, please specify：**______** | | |  |
| 1. Child’s birthday (check birth certificate) | | | _____Year_____Month_____Day | | |  |
| 1. Child’s gender | | | 1=Male， 2=Female | |  |  |
| 1. Child’s ethnicity | | | 1=Han， 2=Mongols，  3=Hui， 4=Other,  please specify | |  |  |
| 1. Child’s birth order | | |  | |  |  |
| 1. Child’s gestational age (check birth certificate) | | | ____weeks____days | | |  |
| 1. Whether the child was preterm birth? | | | 1=yes， 2=no， 3=not sure | |  |  |
| 1. Delivery method | | | 1= natural birth  (go to 8.5)，  2= C-section， 3= not sure | |  |  |
| 8.1 Who chose to do the C-section? | | | 1=doctor， 2= child’s mother，  3= family member | |  |  |
| 8.2 Reason for C-section | | |  | | |  |
| 8.3 Who decided the timing of the C-section? | | | 1=doctor, 2= child’s mother,  3= family member | |  |  |
| 8.4 Was the child’s birthday deliberately chosen? | | | 1=yes 2=no | |  |  |
| 8.5 Did you consider having an early delivery for the purpose of school enrollment? (September 1^st^ is the cutoff for enrollment age） | | | 1= Yes 2=No | |  |  |
| 8.6 Cost of delivery (excluding insurance coverage) | | | 元 | |  |  |
| 8.7 Insurance coverage of the delivery cost | | | 元 | |  |  |
| 1. Delivery location | | | 1=County-level hospital， 2=Township-level hospital，3=family planning committee facility，4=at home，  5=other, please specify，6=not sure | |  |  |
| 1. Child’s birth height | | | Centimeters | |  |  |
| 1. Child’s birth weight | | | Grams | |  |  |
| **2. Infant and Young Child Feeding** | | | | | |  |
| **Question** | | | **Answer Options** | | **Answer** |  |
| 12.When the child first fed breastmilk after birth? | | | 1=Within an hour，2= More than an hour but in the same day，3=Within week，  4=Never，5= After a week | |  |  |
| 12.2 Did the child have any vitamin supplements yesterday? | | | | 1=Yes， 2=No | |  |
| 12.3 Did the child have any other supplements yesterday? | | | | 1=Yes， 2=No | |  |
| 12.0 Has the child ever been breastfed? | | | 1=Yes， 2=No **(go to 15.1)** | |  |  |
| 13.**Was the child breastfed yesterday**？ | | | | 1=Yes， 2=No **(go to 14)** | |  |
| **Question** | | | **Answer Options** | | **Answer** |  |
| 13.1 How many times was the child breastfed yesterday? | | | times | |  |  |
| 14.Was the child exclusively breastfed？ | | | 1=Yes**（go to 21.22）**， 2= No | |  |  |
| 15.How long was the child exclusively breastfed? | | | months | |  |  |
| 15.1 Was the child fed water, juice or soup yesterday? | | | 1= Yes， 2= No | |  |  |
| 16.4 Was the child fed yogurt yesterday? | | | 1= Yes， 2= No**（go to 16.7）** | |  |  |
| 16.5 How many times did the child consume yogurt yesterday? | | | time | |  |  |
| 16.7 Did the child consume any other liquids (including soymilk, meat broth)?  Note: count yes if the child consumed meat yesterday. | | | 1= Yes， 2= No | |  |  |
| 16.9 How many times did the child consume non-formula milk yesterday？ | | | 次 | |  |  |
| 18.Has the child ever been fed formula? | | | 1= Yes，2= No**（go to 21.1）** | |  |  |
| 1. 1 How long has the child been fed formula? | | | months | |  |  |
| 18.3 How many times was the child fed formula yesterday？ | | | times | |  |  |
| 21.1 Did the child eat any staple food yesterday, such as rice porridge, flour porridge, steamed bun or rice? | | | 1= Yes， 2= No | |  |  |
| 21.2 Did the baby eat any yellow or orange food yesterday, such as pumpkin, carrot or red sweet potato？ | | | 1= Yes， 2= No | |  |  |
| 21.3 Did the baby eat any root and stem vegetables yesterday, such as potato, yam, radish, white sweet potato？ | | | 1= Yes， 2= No | |  |  |
| 21.4 Did the baby eat any leafy dark green vegetables yesterday？ | | | 1= Yes， 2= No | |  |  |
| 21.5 Did the baby eat any red or yellow fruits such as persimmon, apricot, watermelon, cantaloupe or tomato yesterday？ | | | 1= Yes， 2= No | |  |  |
| 21.6 Did the baby eat any other fruits or vegetables yesterday？ | | | 1= Yes， 2= No | |  |  |
| 21.7 Did the baby eat any organ meats such as animal liver, kidney or heart yesterday？ | | | 1= Yes， 2= No | |  |  |
| 21.8 Did the baby eat any other meat or meat products (e.g., chicken, duck, pork, beef, lamb, etc.) yesterday? | | | 1= Yes， 2= No | |  |  |
| 21.9 Did the baby eat any eggs yesterday? | | | 1= Yes， 2= No | |  |  |
| 21.10 Did the baby eat any fresh or dried fish, shellfish or seafood yesterday? | | | 1= Yes， 2= No | |  |  |
| 21.11 Did the baby eat any beans, peas, lentils, nuts or seeds yesterday? | | | 1= Yes， 2= No | |  |  |
| 21.12 Did the baby eat any dairy products such as cheese and yoghurt yesterday | | | 1= Yes， 2= No | |  |  |
| 21.13 Did the baby eat any oil (including cooking oil) and meat fat yesterday? | | | 1= Yes， 2= No | |  |  |
| **Question** | | | **Answer Options** | | **Answer** |  |
| 21.14 Did the baby eat any snacks such as biscuits, dessert, candy, chocolate or cake yesterday? | | | 1= Yes， 2= No | |  |  |
| 21.15 Did the baby eat any condiments for flavor, such as salt, chilies, spices, cilantro, ginger, garlic, or fish powder yesterday? | | | 1= Yes， 2= No | |  |  |
| 21.16 Did the child have solid food (such as rice and meat buns), semi-solid food (something thick, such as porridge) or soft food (such as flour puree, fruit puree and vegetable puree) yesterday？ | | | 1= Yes， 2= No | |  |  |
| 21.17 How many times did the child have solid food (such as rice and meat buns), semi-solid food (something thick, such as porridge) or soft food (such as flour puree, fruit puree and vegetable puree) yesterday？ | | | time | |  |  |
| 21.22 Have you received free micronutrient supplement packets from the government？ | | | 1= Yes； 2= No**（go to 31）** | |  |  |
| 21.23 How old was the child when you started to receive micronutrient supplement packets from the government？ | | | months | |  |  |
| 21.24 Till now, how many micronutrient supplement packets have you received from the government? | | | packets | |  |  |
| 21.25 How many nutrient supplement packets received from the government were fed to the child last week? | | | packets | |  |  |
| 21.26 Till now, how many micronutrient supplement packets have you fed to the child? | | | packets | |  |  |
| 31. How much money did you spend on micronutrient supplement for the child last month? | | | yuan | |  |  |
| 32. How much money did you spend on baby formula for the child? | | | yuan | |  |  |
| **3、Maternal and child health information** | | | | | |  |
| **Question** | | **Answer Options** | | | **Answer** |  |
| 45. Have the child had free health check-ups? | | 1= Yes， 2= No， 3= Not sure | | |  |  |
| 45.1 Does the child have the vaccination card?  Note: Vaccination card records vaccination. | | 1= Yes， 2= No**（go to 48）**，  3= Not sure | | |  |  |
| 45.2 Did the child receive vaccine on time? **(Check vaccination card)** | | 1= Yes， 2= No | | |  |  |
| 48.Was the child diagnosed with any serious disease in the health check-ups？ | | 0=Never went to check-ups，  1= Yes， 2= No，  3= Not Sure | | |  |  |
| 49. If yes, what was the disease？（Note: if no disease was found, fill “NO”） | |  | | | |  |
| **4、Child health Information** | | | | | |  |
| **Question** | | **Answer Options** | | | **Answer** |  |
| 1. Did the child have a fever in the last two weeks? | | 1= Yes， 2= No | | |  |  |
| 1. Did the child have a cough in the last two weeks? | | 1= Yes， 2= No | | |  |  |
| 1. Did the child have diarrhea in the last two weeks? | | 1= Yes， 2= No | | |  |  |
| 1. Did the child have digestion problems in the last two weeks? | | 1= Yes， 2= No | | |  |  |
| 1. Did the child have a cold in the last two weeks? | | 1= Yes， 2= No | | |  |  |
| 1. How many times was the child sick in the last two weeks？ | | time | | |  |  |
| 59.1 How many days was the child sick in the last two weeks? | | day | | |  |  |
| 1. How much did you spend on doctors for your child s in the past two weeks? | | yuan | | |  |  |
| 60.1 What was the most serious disease your child had in the last two weeks? | | Please specify | | |  |  |
| 60.2 How much did you spend on the most serious disease your child had in the last two weeks？ | | yuan | | |  |  |
| 60.3 During the interview, was the child sick or uncomfortable？ | | 1= Yes， 2= No | | |  |  |
| 61 What is your view of the county-level family planning committee cadres？ | | 1= Strong favorable view,  2= Favorable view，  3= Neutral,  4= Unfavorable view，  5= Strong unfavorable view,  6= never in touch | | |  |  |
| 62 What is your view of the village-level family planning committee cadres？ | | 1= Strong favorable view,  2= Favorable view，  3= Neutral,  4= Unfavorable view，  5= Strong unfavorable view | | |  |  |
| **五、Other caregiving behaviors and plans** | | | | | |  |
| **问题** | | **选项** | | | **答案** |  |
| 74.1 How many 6-18-month-old children does the child often play with? | | number | | |  |  |
| 74.2 How many 18-30-month-old children does the child often play with? | | number | | |  |  |
| 74.3 How many 30-42-month-old children does the child often play with? | | number | | |  |  |
| 1. How many hours did the you spend with other babies’ caregivers yesterday? | | hours | | |  |  |
| 81.1 Did you use toys to play with the child yesterday？ | | 1= Yes， 2= No | | |  |  |
| 81.2 Did you tell stories to the child yesterday？ | | 1= Yes， 2= No | | |  |  |
| 81.3 Did you tell stories using storybooks to the child yesterday？ | | 1= Yes， 2= No | | |  |  |
| 81.4 Did you sing songs to the child yesterday? | | 1= Yes， 2= No | | |  |  |
| 82.1 How many months did the mother stay home taking care of the child full-time after birth? | | months | | |  |  |
| **Question** | | **Option** | | | **Answer** |  |
| 82.2 How many months did the mother work and take care of the child at the same time since the child birth？ | | months | | |  |  |
| 82.3 What was the income of the mother when working and taking care of the child？（Please fill “0” if did not have income） | | yuan | | |  |  |
| 82.4 How many months did the mother migrate out for work full-time (not with the child) since the child birth？ | | months | | |  |  |
| 82.5 What was the income of the mother when migrating out for work (not with the child) full-time？（Please fill “0” if did not have income） | | yuan | | |  |  |
| 82.6 How old was the child when the mother out-migrated for work for the first time？（Please fill “\” if did not migrate out） | | months | | |  |  |
| 84.Is the mother home now taking care of the child? | | 1= Yes， 2= No | | |  |  |
| 85.1 How many months did the father stay home taking care of the child full-time since the child birth？ | | months | | |  |  |
| 85.2 How many months did the father work and take care of the child at the same time since the child birth？ | | months | | |  |  |
| 85.3 What was the income of the father when working and taking care of the child？（Please fill “0” if did not have income） | | yuan | | |  |  |
| 85.4 How many months did the father migrate out for work full-time (not with the child) since the child birth？ | | months | | |  |  |
| 85.5 What was the income of the father when migrating out for work (not with the child) full-time？（Please fill “0” if did not have income） | | yuan | | |  |  |
| 85.6 How old was the child when the father migrated out for work for the first time？（Please fill “\” if did not migrate out） | | months | | |  |  |
| 85.7 Is the father home now taking care of the child? | | 1= Yes， 2= No | | |  |  |
| 85.8 How many family members are farming or doing non-farm business？ | | number | | |  |  |
| 85.9 What is the farming income of the family this year？ | | yuan | | |  |  |
| 85.10 What is the non-farming income of the family this year？ | | yuan | | |  |  |
| **6、Information sources of caregiving knowledge** | | | | | |  |
| **Question** | | | **Option** | | **Answer** |  |
| 1. From whom did you learn your parenting knowledge? (choose all applies) | | | 1=Family, 2=Friends, 3=Village doctor, 4=Birth Control Unit, 5= Director of women’s issues, 6=Other health specialists, 7=Books, 8=TV, 9=Internet, 10=Other, please explain | |  |  |
| 1. Did you ever receive parenting training? | | | 1=Yes, 2=No, 3=Do not know | |  |  |
| 1. In the last year, has anyone instructed you on how to teach your to stay away from dangerous items or places (ex: pesticides, ponds, fires or sockets)? | | | | 1=Yes, 2=No, 3=Do not know |  |  |
| 1. In the last year, has anyone talked to you about which tasks (ex: washing and dressing) the child should be able to perform independently? | | | | 1=Yes, 2=No, 3=Do not know |  |  |
| 1. In the last year, has anyone talked to you about the vocabulary that your child should understand and be able to use? | | | | 1=Yes, 2=No, 3=Do not know |  |  |
| 1. In the last year, has anyone talked to you about the importance of reading storybooks to your child? | | | | 1=Yes, 2=No, 3=Do not know |  |  |
| 1. In the last year, has anyone talked to you about reading storybooks to your children? | | | | 1=Yes, 2=No, 3=Do not know |  |  |
| 1. In the last year, has anyone talked to you about singing songs with your child? | | | | 1=Yes, 2=No, 3=Do not know |  |  |
| 1. In the last year, has anyone talked to you about playing games with your child？ | | | | 1=Yes, 2=No, 3=Do not know |  |  |
| 1. In the last year, has anyone talked to you about how children learn to get along with their peers? | | | | 1=Yes, 2=No, 3=Do not know |  |  |
| 1. In the last year, has anyone talked to you about how to help your children understand and obey rules? | | | | 1=Yes, 2=No, 3=Do not know |  |  |
| **7、Caregiving environment** | | | | | |  |
| **Question** | | **Option** | | | **Answer** |  |
| 1. Does the child have access to an independent play area at home (such as a cushioned area)? | | 1=Always, 2=Often, 3=Sometimes, 4=Once in a while, 5=Never | | |  |  |
| 1. Is there cellphone signal in your home? | | 1= Yes, 2= No | | |  |  |
| 1. Does any family member smoke cigarettes? | | 1= Yes，2= No (**go to 102.1**） | | |  |  |
| 1. If a family member smokes, does he/she smoke at home? | | 1= Never，2= Sometimes，3= Often | | |  |  |
| 102.1 How many siblings does the child’s father have? | | number | | |  |  |
| 102.2 How many of the father’s siblings live in the same village? | | number | | |  |  |
| 102.3 How many of the father’s siblings have out-migrated for work? | | number | | |  |  |
| 102.4 How many paternal cousins (children of fathers’ siblings) does the child have? | | number | | |  |  |
| 102.5 How many paternal cousins (children of fathers’ siblings) live in the same village? | | number | | |  |  |
| 102.6 How many paternal cousins (children of fathers’ siblings) are under 3 years old? | | number | | |  |  |
| 102.7 How many siblings does the child’s mother have? | | number | | |  |  |
| 102.8 How many of the mother’s siblings live in the same village? | | number | | |  |  |
| 102.9 How many of the mother’s siblings have out-migrated for work? | | number | | |  |  |
| 102.10 How many maternal cousins (children of mothers’ siblings) does the child have? | | number | | |  |  |
| 102.11 How maternal cousins (children of mothers’ siblings) live in the same village? | | number | | |  |  |
| 102.12 How many maternal cousins (children of mothers’ siblings) are under 3 years old? | | number | | |  |  |
| 102.13 Do family members get along well with each other? | | 1= Yes, 2= No; | | |  |  |
| 102.14 Do family members sincerely support each other? | | 1=是，2=否 | | |  |  |
| 102.15 Do family members blame or criticize each other？ | | 1= Always ，2= Sometimes ，3= Never | | |  |  |
| 102.16 When family members have trouble with each other, do they argue loudly？ | | 1= Always ，2= Sometimes ，3= Never | | |  |  |
| 102.17 Do family members smash things when angry？ | | 1= Always ，2= Sometimes ，3= Never | | |  |  |
| 102.18 Do family members beat others when angry？ | | 1= Always ，2= Sometimes ，3= Never | | |  |  |
| **8. Parenting behavior** | | | | | |  |
| **问题** | | | **选项** | | **答案** |  |
| 111. In the last two days, how many times did you hug or kiss your child or use other ways to express intimate feelings? | | | Times | |  |  |
| 112. How much time does the child spend watching TV or videos each day on average? | | | Minutes (fill ”0” if none) | |  |  |
| 113. How much time does the child spend playing alone each day on average? | | | Minutes (fill ”0” if none) | |  |  |
| 113.2 **How long did you (or other caregivers) spend playing with the child by yourself yesterday**? | | | Minutes (fill ”0” if none) | |  |  |
| 113.3 **How long did you (or other caregivers) tell stories to the child by yourself yesterday**? | | | Minutes (fill ”0” if none) | |  |  |
| 113.4 **How long did you (or other caregivers) tell stories using storybook to the child by yourself yesterday**? | | | Minutes (fill ”0” if none) | |  |  |
| 113.5 **How long did you (or other caregivers) sing songs to the child yesterday by yourself yesterday**? | | | Minutes (fill ”0” if none) | |  |  |
| 113.6 **How many times did the child cry yesterday?** | | | Times | |  |  |
| 113.7 How many times did the child wet his/her pants yesterday? | | | Times | |  |  |
| 113.8 How many times did the child stain his/her clothes with stool **yesterday**？ | | | Times | |  |  |
| 113.9 How many times did the child get angry yesterday？ | | | Times | |  |  |
| 113.10 How many times did you (or other caregivers) **scold the child yesterday**？ | | | Times | |  |  |
| 113.11 **How many times did you (or other caregivers) spank the child yesterday?** | | | Times | |  |  |
| 113.12 **How many times did the child fall down yesterday?** | | | Times | |  |  |
| 113.13 How many times did the child get into trouble with other children yesterday? | | | Times | |  |  |
| 113.14 Does the child curse? | | | 1= Yes，2= No | |  |  |
| 113.15 Does the child spit on others? | | | 1= Yes，2= No | |  |  |
| 113.16 Does the child hit others? | | | 1= Yes，2= No | |  |  |
| 113.17 Does the child hit others with objects? | | | 1= Yes，2= No | |  |  |
| 113.18 **How much money did you spend on toys for your child** in the last year? | | | yuan | |  |  |
| 113.19 **How much money did you spend on** books for your child in the last year? | | | yuan | |  |  |
| 114. Do you raise her tone or yell at your child when parenting? | | | 1=Often, 2=Sometimes, 3=Rarely, 4=Never, 5=Do not know | |  |  |
| 115. Do you physically punish your child when parenting? | | | 1=Often, 2=Sometimes, 3=Rarely, 4=Never, 5=Do not know | |  |  |
| 116.Do you take away your child’s toys or the things that he/she wants as a method of parenting? | | | 1=Often, 2=Sometimes, 3=Rarely, 4=Never, 5=Do not know | |  |  |
| 117.Do you set time limits to cut off your child’s activities (such as watching TV or playing games) as a method of parenting? | | | 1=Often, 2=Sometimes, 3=Rarely, 4=Never, 5=Do not know | |  |  |
| 118.Do you explain to your child why his/her actions are inappropriate when parenting? | | | 1=Often, 2=Sometimes, 3=Rarely, 4=Never, 5=Do not know | |  |  |
| **9、Caregiver’s parenting belief** | | | | | |  |
| **Question** | **Option** | | | | **Answer** |  |
| 1. Thinking about this past month, what is your opinion of the phrase "I really enjoyed being with my child." | 1=Completely incorrect, 2=Mostly incorrect, 3=Half correct, 4=Mostly correct, 5=Completely correct | | | |  |  |
| 1. Thinking about this past month, what is your opinion of the phrase "I get along with my child." | 1=Completely incorrect, 2=Mostly incorrect, 3=Half correct, 4=Mostly correct, 5=Completely correct | | | |  |  |
| 1. Thinking about this past month, what is your opinion of the phrase "I am annoyed when I am with my child." | 1=Completely incorrect, 2=Mostly incorrect, 3=Half correct, 4=Mostly correct, 5=Completely correct | | | |  |  |
| 1. Thinking about this past month, what is your opinion of the phrase "I am nervous (stressed) while I'm with my child." | 1=Completely incorrect, 2=Mostly incorrect, 3=Half correct, 4=Mostly correct, 5=Completely correct | | | |  |  |
| 1. Thinking about this past month, what is your opinion of the phrase "I am always ignored by my child when talking to him.” | 1=Completely incorrect, 2=Mostly incorrect, 3=Half correct, 4=Mostly correct, 5=Completely correct | | | |  |  |
| 1. Thinking about this past month, what is your opinion of the phrase "I do not know how to communicate with my child from his perspective”. | 1=Completely incorrect, 2=Mostly incorrect, 3=Half correct, 4=Mostly correct, 5=Completely correct | | | |  |  |
| 1. Thinking about this past month, what is your opinion of the phrase "I think it is fun to play games with my child” | 1=Completely incorrect, 2=Mostly incorrect, 3=Half correct, 4=Mostly correct, 5=Completely correct | | | |  |  |
| 1. I think that playing with my child is important. | 1=Completely incorrect, 2=Mostly incorrect, 3=Half correct, 4=Mostly correct, 5=Completely correct | | | |  |  |
| 1. What is your opinion of the phrase "I know how to play with my child.” | 1=Completely incorrect, 2=Mostly incorrect, 3=Half correct, 4=Mostly correct, 5=Completely correct | | | |  |  |
| 1. I think that reading books or telling stories to my child are important. | 1=Completely incorrect, 2=Mostly incorrect, 3=Half correct, 4=Mostly correct, 5=Completely correct | | | |  |  |
| 1. What is your opinion of the phrase “I know how to read a storybook with my child.” | 1=Completely incorrect, 2=Mostly incorrect, 3=Half correct, 4=Mostly correct, 5=Completely correct | | | |  |  |
| 1. What is your opinion of the phrase “It is a caregiver’s responsibility to help the child understand the world around him/her” | 1=Completely incorrect, 2=Mostly incorrect, 3=Half correct, 4=Mostly correct, 5=Completely correct | | | |  |  |
| 1. I think my child’s performance in school is important. | 1=Completely incorrect, 2=Mostly incorrect, 3=Half correct, 4=Mostly correct, 5=Completely correct | | | |  |  |
| **10、Family characteristics** | | | | | |  |
| **Question** | | | **Option** | | **Answer** |  |
| 133.What is the distance from your home to the village committee? | | | Meters | |  |  |
| 134.Does your family get subsistence allowances? | | | 1= Yes， 2= No | |  |  |
| 135.What is your family yearly income? | | | yuan | |  |  |
| 136.What is the construction are of your house？ | | | square meters | |  |  |
| 137.What is the value of your house? | | | 1=10k and under, 2=10k-50k, 3=50k-100k, 4=100k-300k, 5=300k and above | |  |  |
| 138.Does your home have tap water？ | | | 1= Yes， 2= No | |  |  |
| 139.Does your home have flush toilets? | | | 1= Yes， 2= No | |  |  |
| 140.Does your home have a boiler? | | | 1= Yes， 2= No | |  |  |
| 141.Does your home have a washing machine? | | | 1= Yes， 2= No | |  |  |
| 142.Does your home have computer? | | | 1= Yes， 2= No | |  |  |
| 143.Does your home have access to the Internet? | | | 1= Yes， 2= No | |  |  |
| 144.Does your home have a refrigerator? | | | 1= Yes， 2= No | |  |  |
| 145.Does your home have an air conditioner? | | | 1= Yes， 2= No | |  |  |
| 146.Does your home have a motorbike or electric bike? | | | 1= Yes， 2= No | |  |  |
| 147.Does your home have a car or van? | | | 1= Yes， 2= No | |  |  |
| 148.Did the mother of child out-migrate for work? | | | 1= Yes， 2= No (end) | |  |  |
| 149.If the mother migrated out for work, did she work as nanny? | | | 1= Yes， 2= No | |  |  |
| 150. Did the mother learn or pay attention to how urban people take care of their children? | | | 1= Yes， 2= No | |  |  |
| 151.Would you be willing to be the manager of a parenting center for a compensation of 1000 yuan? | | | 1= Yes， 2= No | |  |  |
| 152. Would you be willing to be the manager of a parenting center for a compensation of 1500 yuan? | | | 1= Yes， 2= No | |  |  |
