# Supplementary material for: Correlates of participation in community-based interventions: Evidence from a parenting program in rural China
Source: PLoS One. 2020 Sep 8;15(9):e0238841. doi: 10.1371/journal.pone.0238841 (PMC7478867; doi:10.1371/journal.pone.0238841)
Supplement: S3 File — (DOCX) [file pone.0238841.s003.docx]

**婴幼儿养育中心项目问卷（由第一看护人填写）**

**陕西省______市______县______镇______ 村**

**家庭编码：____________**

**婴儿姓名：____________**

**父亲姓名：____________**

**母亲姓名：____________**

**受访人姓名：_______**

**受访者电话：____________**

**是否是第一看护人：是/否**

**调查员编码：_____________**

| 一、交通工具 | | | | |
| --- | --- | --- | --- | --- |
| 有关以下的交通工具： | 1.这个交通工具您平均多久用一次？（1=一天多次、2=一天一次、3=一周多次、4=一周一次、5=一个月一次或更少） | 2.一般您用这个交通工具的时候需要有人陪同吗？（1=是；0=否）  0🡪 跳到第4题 | 3.谁陪同？  （1=宝宝爸爸；2=妈妈；3=爷爷；4=奶奶；5=其他，请说明） | 4.使用这个交通工具时带上孩子一起方便吗？（1=是、0=否） |
| 1. 步行 |  |  |  |  |
| 1. 自行车 |  |  |  |  |
| 1. 电动车/摩托车 |  |  |  |  |
| 1. 班车/公交车 |  |  |  |  |
| 1. 私家车 |  |  |  |  |
| 1. 其他，请说明 |  |  |  |  |

| 问题 | 选项 | 答案 |
| --- | --- | --- |
| 5.您觉得去养育中心有多方便，或有多不方便？ | 1=非常方便、2=比较方便、3=一般、4=不太方便、5=非常不方便 |  |
| 6.去养育中心方不方便会不会影响您去养育中心的意愿？ | 1=是、0=否 |  |

| 二、日常活动 | | |
| --- | --- | --- |
| 7. 您去养育中心一般怎么去？（用什么样的交通方式？） | 1=步行、2=自行车、3=电动车/摩托车、4=班车/公交车、5=私家车、6=其他，请说明 |  |
| 8. 去养育中心路上花多长时间？ | （分钟） |  |
| 9. 去养育中心的路雨天通吗？ | 1=是； 0=否 |  |
| 10. 去养育中心的路冬天下雪天通吗？ | 1=是； 0=否 |  |
| 11. 你们家去田地里路上花多长时间？ | （分钟） |  |
| 12.您家农忙一般是什么时候？ | （月）阳历 |  |
| 13.农忙时， 您一般一天花几个小时在农活上？（问第一负责人） | （小时） |  |
| 14. 农闲时，您一般一天花几个小时在农活上？（问第一负责人） | （小时） |  |
| 15. 除了养育中心外， 你们村上有没有适宜儿童的公共休闲场所？（公园、广场、儿童娱乐设施等） | 1=是；0=否 |  |
| 16. 您多久去一次村上的公共休闲场所？ | 1=一天多次、2=一天一次、3=一周多次、4=一周一次、5=一个月一次或更少、6=没去过 |  |

| 三、社交情况基本信息(由第一看护人回答) | | | | | | | |  |
| --- | --- | --- | --- | --- | --- | --- | --- | --- |
| 本村其他参加养育项目的家长和婴儿 | | | | 在宝宝去养育中心的头一年( 期间)，你和以下这个家庭的任意家庭成员交流的情况，比如聊家长里短，宝宝的健康发育状况？(0=没来往、1=几月一次、2=一月一次、3=一周一次、4=一周两三次、 5=每天) | 在宝宝去养育中心之前，你和这个家庭的任何一个家庭成员有交流吗，比如聊家长里短，宝宝的健康发育状况？(0=没来往、1=几月一次、2=一月一次、3=一周一次、4=一周两三次、5=每天) | 你们家到他们家用最常用的交通工具要多久  (分钟) | 你们家到他们家的距离有多远？(米） | 你们两家是亲戚  (远房亲戚)吗？(1=是、0=否) |
| 家庭编号 | 婴儿母亲  /父亲姓名 | 婴儿第一  负责人姓名 | 婴儿姓名  (小名) |  |  |  |  |  |
|  |  |  |  |  |  |  |  |  |
|  |  |  |  |  |  |  |  |  |
|  |  |  |  |  |  |  |  |  |
|  |  |  |  |  |  |  |  |  |
|  |  |  |  |  |  |  |  |  |
|  |  |  |  |  |  |  |  |  |
|  |  |  |  |  |  |  |  |  |
|  |  |  |  |  |  |  |  |  |
|  |  |  |  |  |  |  |  |  |
|  |  |  |  |  |  |  |  |  |
|  |  |  |  |  |  |  |  |  |
|  |  |  |  |  |  |  |  |  |
|  |  |  |  |  |  |  |  |  |
|  |  |  |  |  |  |  |  |  |
|  |  |  |  |  |  |  |  |  |
|  |  |  |  |  |  |  |  |  |
|  |  |  |  |  |  |  |  |  |
|  |  |  |  |  |  |  |  |  |
|  |  |  |  |  |  |  |  |  |
|  |  |  |  |  |  |  |  |  |
|  |  |  |  |  |  |  |  |  |
|  |  |  |  |  |  |  |  |  |
|  |  |  |  |  |  |  |  |  |
|  |  |  |  |  |  |  |  |  |
|  |  |  |  |  |  |  |  |  |
|  |  |  |  |  |  |  |  |  |
|  |  |  |  |  |  |  |  |  |
|  |  |  |  |  |  |  |  |  |
|  |  |  |  |  |  |  |  |  |
|  |  |  |  |  |  |  |  |  |
|  |  |  |  |  |  |  |  |  |
|  |  |  |  |  |  |  |  |  |
|  |  |  |  |  |  |  |  |  |
|  |  |  |  |  |  |  |  |  |

| 四、家庭成员外出情况 | | | |
| --- | --- | --- | --- |
| 问题 |  | 选项 | 回答 |
| 在宝宝去养育中心的头一年（__________期间） | 17.你们主要在本村养育宝宝吗？ | 1=是、0=否（0🡪跳到第24题） |  |
|  | 18.你们是否有偶尔带宝宝外出？ | 1=是、0=否（0🡪跳到第20题） |  |
|  | 19.如果你们偶尔有带宝宝出门，宝宝不在本村的时间有多长？ | （月） |  |
|  | 20.爸爸不在家里住时间多长？ | （月） |  |
|  | 21.妈妈不在家里住时间多长？ | （月） |  |
|  | 22.爷爷（外公）不在家里住时间多长？ | （月） |  |
|  | 23.奶奶（外婆）不在家里住时间多长？ | （月） |  |
|  | 24.如果你们不在本村养育宝宝，那宝宝平时在哪里呢？ | 1=本乡内、2=本县非本乡、3=本省非本县、4=外省 |  |
|  | 25.如果你们不在本村养育宝宝，那宝宝在村里时间有多久？ | （月） |  |

| 五、婴幼儿兄弟姐妹情况 | | | | | | | | | | |
| --- | --- | --- | --- | --- | --- | --- | --- | --- | --- | --- |
| （孩子没有兄弟姐妹跳过） | | | | | | | | | | |
| 问题 | 选项（兄弟姐妹 1） | 答案 | 选项（兄弟姐妹 2） | | | | 答案 | 选项（兄弟姐妹3） | | 答案 |
| 宝宝的兄弟姐妹是： | 1=哥哥、2=弟弟、3=姐姐、4=妹妹 |  | 1=哥哥、2=弟弟、3=姐姐、4=妹妹 | | | |  | 1=哥哥、2=弟弟、3=姐姐、4=妹妹 | |  |
| 26. 该婴幼儿的兄弟姐妹生日？ | 年/月/日 |  | 年/月/日 | | | |  | 年/月/日 | |  |
| **在宝宝能去养育中心的头一年（_______________期间）** | | | | | | | | | | |
| 27.这个兄弟姐妹在读什么学校？ | 1=幼儿园／托儿所、2=小学、3=初中或以上、 4=不上学（4跳到33题） |  | 1=幼儿园／托儿所、2=小学、3=初中或以上、4=不上学（4跳到第33题） | | | |  | 1=幼儿园／托儿所、2=小学、3=初中或以上、 4=不上学（4跳到第33题） | |  |
| 28.这个兄弟姐妹上学的地点是否和该幼儿在同一个村？ | 1=是、0=否 （1🡪跳到30题） |  | 1=是、0=否  （1🡪跳到30题） | | | |  | 1=是、0=否  （1🡪跳到30题） | |  |
| 29.兄弟姐妹学校所在的村/镇 | 村或者镇的名字 |  | 村或者镇的名字 | | | |  | 村或者镇的名字 | |  |
| 30.您是否负责接送宝宝的兄弟姐妹上学？（0🡪跳到第六部分） | 1=是、0=否 |  | 1=是、0=否 | | | |  | 1=是、0=否 | |  |
| 31.您多久去一次兄弟姐妹的学校？ | 1=一天多次、2=一天一次、3=一周多次、4=一周一次、5=一个月一次或更少 |  | 1=一天多次、2=一天一次、3=一周多次、4=一周一次、5=一个月一次或更少 | | | |  | 1=一天多次、2=一天一次、3=一周多次、4=一周一次、5=一个月一次或更少 | |  |
| 32.去孩子兄弟姐妹的学校路上花多长时间？ | 分钟 |  | 分钟 | | | |  | 分钟 | |  |
|  |  |  |  | | | |  |  | |  |
|  |  |  |  | | | |  |  | |  |
| 六、 家庭成员健康状况 | | | | | | | | | | |
| 问题 | | | | | 选项 | | | | 答案 | |
| **在宝宝能去养育中心的头一年（_______________期间）** | | | | | | | | | | |
| 之前奶奶（外婆）的身体状况 ­­­­______________ | | | | 好 或者 空 （跳到35题）；不太好 继续 | | | | | | |
| 33.奶奶（外婆）需要家人照顾吗？ | | | | 1=是、0=否（0🡪跳到35题） | | | | |  | |
| 34.家中照顾奶奶（外婆）的人是不是该婴幼儿的第一看护人？ | | | | 1=是、0=否、2=奶奶（外婆）是主要看护人 | | | | |  | |
| 之前爷爷（外公）的身体状况 _______________ | | | | 好 或者 空 ( 跳到37题)；不太好 继续 | | | | | | |
| 35爷爷（外公）需要家人照顾吗？ | | | | 1=是、0=否（0🡪跳到37题） | | | | |  | |
| 36. 家中照顾爷爷（外公）的人是不是该婴幼儿的第一看护人？ | | | | 1=是、0=否、2爷爷（外公）是主要看护人 | | | | |  | |
| 七、 离村安全隐患 | | | | | | | | | | |
| 问题 | | | | | | 选项 | | | | 答案 |
| **在宝宝能去养育中心的头一年（_______________期间）** | | | | | | | | | | |
| 37. 你从家到养育中心，是否需要离开自己所在的自然村？ | | | | | | 1=是、0=否 | | | |  |
| 38. 你带着该婴幼儿去养育中心，路上安全情况如何？ | | | | | | 1=非常危险、2=比较危险、3=一般、4=比较安全、5=非常安全 | | | |  |
| 39. 其他家庭成员对你带着孩子去养育中心是否会存在担心？ | | | | | | 1=是、0=否 | | | |  |
