# Supplementary material for: Correlates of participation in community-based interventions: Evidence from a parenting program in rural China
Source: PLoS One. 2020 Sep 8;15(9):e0238841. doi: 10.1371/journal.pone.0238841 (PMC7478867; doi:10.1371/journal.pone.0238841)
Supplement: S4 File — (DOCX) [file pone.0238841.s004.docx]

**Nourish the Future Program Caregiver Survey (to be answered by the main caregivers）**

**____Province ____City ____County ____Township ____Village**

**Household ID：____________**

**Child name：____________**

**Father name：____________**

**Mother name：____________**

**Interviewee name：_______**

**Interviewee contact：____________**

**Whether the interviewee is the primary caregiver：yes/ no**

**Interviewer ID：_____________**

| 1、Transportation | | | | |
| --- | --- | --- | --- | --- |
| Regarding the following means of transportation | 1. How often you take this transportation?  1= a few times per day; 2= once per day; 3 = a few times per week; 4= once per month; 5= fewer than once per month | 2. Do you need the help of others when using this transportation?  1= Yes; 0= No (go to 4) | 3. Who accompanies you? (relationship to child)  1= father；2= mother；3= grandfather；4=grandmother；5= other, please specify | 4. Is it convenient to use this transportation with your child?  1= Yes, 2= No |
| 1. Walking |  |  |  |  |
| 1. Bicycle |  |  |  |  |
| 1. Motorized   bicycle |  |  |  |  |
| 1. Shuttle bus |  |  |  |  |
| 1. Car or van |  |  |  |  |
| 1. Other, please specify |  |  |  |  |

| Question | Option | Answer |
| --- | --- | --- |
| 5. Whether you find it convenient to go to the parenting center? | 1= Very convenient, 2= Somewhat convenient, 3= Neutral, 4= Somewhat inconvenient, 5=Very inconvenient |  |
| 6. How does the convenience of going to the parenting center affect your decision to go to the parenting center? | 1= Yes, 0= No |  |

| 2、Daily routine information | | |
| --- | --- | --- |
| 7. How do you usually go to the parenting center? (by what means of transportation) | 1= Walking, 2= Bicycle, 3= Motorized bicycle, 4= Shuttle bus, 5=Car or van, 6= other, please specify |  |
| 8. How long it takes to get to the parenting center? | minutes |  |
| 9. Is the road to the parenting center accessible during rainy days? | 1= Yes, 0= No |  |
| 10. Whether is road to the parenting center is accessible during in the snow? | 1= Yes, 0= No |  |
| 11. How long does it take to get to your farmland? | minutes |  |
| 12. When is the usual busy farming season in your area? | month (in solar calendar) |  |
| 13. During the busy farming season, how long do you usually spend on farming? (to be answered by the primary caregiver) | hours |  |
| 14. During the slack farming season, how long do you usually spend on farming? (to be answered by the primary caregiver) | hours |  |
| 15. Beside the parenting center, are there other public facilities that are suitable for the child to play? (e.g., park, playground, etc.) | 1= Yes, 0= No |  |
| 16. How often do you go to public facilities for leisure purposes? | 1= A few times per day, 2= Once per day, 3= A few times per day, 4= Once per month, 5= Fewer than once per month, 6= Never |  |

| 3、Social network information (to be answered by the primary caregiver) | | | | | | | |  |
| --- | --- | --- | --- | --- | --- | --- | --- | --- |
| Caregivers and their children in eligible households in the same village | | | | During the first year of operation of the parenting center, how often did you interact with any household member in the following households (not limited to topics about children and parenting)?  0= Never, 1= Once every a few months, 2= Once per month, 3= Once per week, 4= two/three times per week, 5= everyday | Before going to the parenting center, how often did you interact with any household members in the following households, (not limited to topics about children and parenting)?  0= Never, 1= Once every a few months, 2= Once per month, 3= Once per week, 4= two/three times per week, 5= everyday | How long does it take to get to their home from your home taking the most commonly used transportation (minutes) | How far is their home from your home? (meters) | Are you relatives?  1= Yes 0= No |
| Household ID | Name of the father/ mother of the child | Name of the primary caregiver | Name of the child |  |  |  |  |  |
|  |  |  |  |  |  |  |  |  |
|  |  |  |  |  |  |  |  |  |
|  |  |  |  |  |  |  |  |  |
|  |  |  |  |  |  |  |  |  |
|  |  |  |  |  |  |  |  |  |
|  |  |  |  |  |  |  |  |  |
|  |  |  |  |  |  |  |  |  |
|  |  |  |  |  |  |  |  |  |
|  |  |  |  |  |  |  |  |  |
|  |  |  |  |  |  |  |  |  |
|  |  |  |  |  |  |  |  |  |
|  |  |  |  |  |  |  |  |  |
|  |  |  |  |  |  |  |  |  |
|  |  |  |  |  |  |  |  |  |
|  |  |  |  |  |  |  |  |  |
|  |  |  |  |  |  |  |  |  |
|  |  |  |  |  |  |  |  |  |
|  |  |  |  |  |  |  |  |  |
|  |  |  |  |  |  |  |  |  |
|  |  |  |  |  |  |  |  |  |
|  |  |  |  |  |  |  |  |  |
|  |  |  |  |  |  |  |  |  |
|  |  |  |  |  |  |  |  |  |
|  |  |  |  |  |  |  |  |  |
|  |  |  |  |  |  |  |  |  |
|  |  |  |  |  |  |  |  |  |
|  |  |  |  |  |  |  |  |  |
|  |  |  |  |  |  |  |  |  |
|  |  |  |  |  |  |  |  |  |
|  |  |  |  |  |  |  |  |  |
|  |  |  |  |  |  |  |  |  |
|  |  |  |  |  |  |  |  |  |
|  |  |  |  |  |  |  |  |  |
|  |  |  |  |  |  |  |  |  |

| 4、Family out-migration information | | | |
| --- | --- | --- | --- |
|  | Question | Option | Answer |
| During the first year of operation of the parenting center | 17. Did you raise the baby in the village? | 1= Yes, 0=No (go to 24) |  |
|  | 18. Did you occasionally out-migrate with the child? | 1= Yes, 0=No (go to 20) |  |
|  | 19.If you occasionally out-migrated with the child, how long did the child live outside the village? | months |  |
|  | 20. How long was the father not living at home? | months |  |
|  | 21. How long was the mother not living at home? | months |  |
|  | 22. How long was the grandfather not living at home? | months |  |
|  | 23. How long was the mother not living at home? | months |  |
|  | 24. If you did not raise the child in the village, where did you raise the child? | 1= in this township, 2= in a different townshipin this county, 3=in a different county in this province, 4= outside of this province |  |
|  | 25. If you did not raise the child in the village, how long did the child spend in the village | months |  |

| 5、Sibling information | | | | | | | | | |
| --- | --- | --- | --- | --- | --- | --- | --- | --- | --- |
| (Go to the next section if the child does not have sibling) | | | | | | | | | |
| Question | Option (sibling 1) | Answer | Option (sibling 2) | | | Answer | Option (sibling 3) | | Answer |
| The relationship of the sibling to the child | 1= elder brother,  2= younger brother,  3= elder sister,  4= younger sister |  | 1= elder brother,  2= younger brother,  3= elder sister,  4= younger sister | | |  | 1= elder brother,  2= younger brother,  3= elder sister,  4= younger sister | |  |
| 26. The birthday of the sibling | year/month/day |  | year/month/day | | |  | year/month/day | |  |
| During the first year of operation of the parenting center | | | | | | | | | |
| 27. What school did the sibling go to? | 1= Preschool/ kindergarten,  2= Primary school,  3=Junior school and above,  4=not going to school (go to next section) |  | 1= Preschool/ kindergarten,  2= Primary school,  3=Junior school and above,  4=not going to school (go to next section) | | |  | 1= Preschool/ kindergarten,  2= Primary school,  3=Junior school and above,  4=not going to school (go to next section) | |  |
| 28. Is the sibling’s school in the same village? | 1= Yes (go to 30), 0= No |  | 1= Yes (go to 30),  0= No | | |  | 1= Yes (go to 30),  0= No | |  |
| 29. Name of the township/ village that the school is located in | village/ township name |  | village/ township name | | |  | village/ township name | |  |
| 30. Are you responsible for drop-off/pickup of the sibling from school? | 1= Yes, 0= No (go to next section) |  | 1= Yes, 0= No (go to next section) | | |  | 1= Yes, 0= No (go to next section) | |  |
| 31. How often do you go to the sibling’s school? | 1= A few times per day,  2= Once per day,  3= A few times per week,  4= Once per week,  5=Fewer than once per month |  | 1= A few times per day,  2= Once per day,  3= A few times per week,  4= Once per week,  5=Fewer than once per month | | |  | 1= A few times per day,  2= Once per day,  3= A few times per week,  4= Once per week,  5=Fewer than once per month | |  |
| 32. How long does it take to go to the sibling’s school? | minutes |  | minutes | | |  | minutes | |  |
|  |  |  |  | | |  |  | |  |
|  |  |  |  | | |  |  | |  |
| 6、 Family member health information | | | | | | | | | |
| Question | | | | | Option | | | Answer | |
| During the first year of operation of the parenting center | | | | | | | | | |
| Grandmother’s health condition is (prefilled) | | | | If “Good” or “NA” （go to 35）, if “Not good” (go to 33) | | | | | |
| 33. Does the grandmother need to be taken care of? | | | | 1=是、0=否 (0 go to 35) | | | |  | |
| 34. Does the primary caregiver of the child also take care of the grandmother? | | | | 1= Yes, 0= No, 2= Grandmother is the caregiver of the child | | | |  | |
| Grandfather’s health condition is (prefilled) | | | | If “Good” or “NA” （go to 37）, if “Not good” (go to 35) | | | | | |
| 35. Does the Grandfather need to be taken care of? | | | | 1=是、0=否 (0 go to 37) | | | |  | |
| 36. Does the primary caregiver of the child also take care of the grandfather? | | | | 1= Yes, 0= No, 2= Grandfather is the caregiver of the child | | | |  | |
| 7、 Safety concern information | | | | | | | | | |
| Question | | | | | Option | | | Answer | |
| During the first year of operation of the parenting center | | | | | | | | | |
| 37. To travel from your home to the parenting center, do you need to leave your own village group? | | | | | 1= Yes, 0= No | | |  | |
| 38. When you take your child to the parenting center, how safe is the road to the parenting center? | | | | | 1= Very dangerous, 2= Somewhat dangerous, 3= Neutral, 4= Somewhat safe, 5= Very safe | | |  | |
| 39. Do other family members worry about you taking the child to the parenting center? | | | | | 1= Yes, 0= No | | |  | |
