# Supplementary figures and images for: Correlates of participation in community-based interventions: Evidence from a parenting program in rural China
Source: PLoS One. 2020 Sep 8;15(9):e0238841. doi: 10.1371/journal.pone.0238841 (PMC7478867; doi:10.1371/journal.pone.0238841)

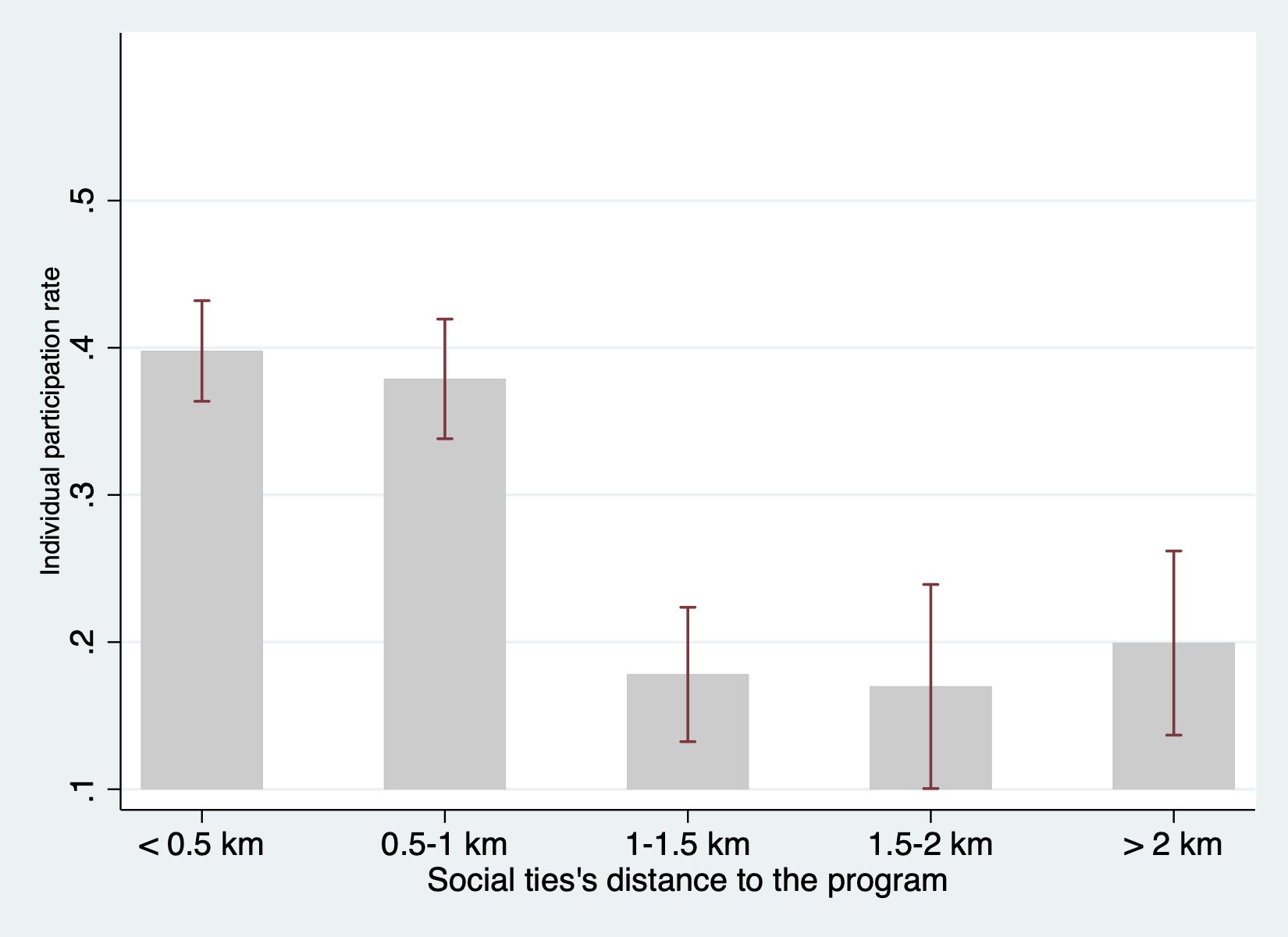

Supplement: S1 Fig — Source: Authors’ Survey. (TIF) [file pone.0238841.s005.tif]
