# Supplementary material for: Correlates of participation in community-based interventions: Evidence from a parenting program in rural China
Source: PLoS One. 2020 Sep 8;15(9):e0238841. doi: 10.1371/journal.pone.0238841 (PMC7478867; doi:10.1371/journal.pone.0238841)
Supplement: S1 Appendix — (DOCX) [file pone.0238841.s006.docx]

**S1 Appendix. Analysis of Attrition.**

In the second survey round, we were only able to follow-up with 682 of the 819 households in our study sample, resulting in a 16.7% attrition rate. To test for attrition bias, we conduct a linear multivariate regression analysis examining how child and household characteristics correlate to attrition. We use a dummy variable that equals to one if we were not able to interview the household during the second-round survey as the dependent variable. The results of this analysis are presented in S1 Table.

We find that number of friends and whether either grandparent is the primary caregiver are significantly correlated with attrition. Number of friends is negatively associated with attrition, meaning that caregivers with more friends were less likely to attrit. Specifically, with one additional friend, the likelihood of attrition decreased by 4.6%, p<0.001. For children whose either grandparent is the primary caregiver, the probability of attrition decreased by 7.1%, p<0.05.
