# Supplementary material for: Correlates of participation in community-based interventions: Evidence from a parenting program in rural China
Source: PLoS One. 2020 Sep 8;15(9):e0238841. doi: 10.1371/journal.pone.0238841 (PMC7478867; doi:10.1371/journal.pone.0238841)
Supplement: S1 Table — (DOCX) [file pone.0238841.s007.docx]

**S1 Table. Correlates of attrition.**

|  | Attrition |
| --- | --- |
| Number of social ties | -0.046*** |
|  | (0.009) |
| Child age (months) | -0.003 |
|  | (0.002) |
| Male child | 0.051 |
|  | (0.028) |
| Standardized BSID-III Cognitive Score | -0.003 |
|  | (0.014) |
| Only child | -0.009 |
|  | (0.028) |
| Grandparent is primary caregiver | 0.071* |
|  | (0.029) |
| Primary caregiver has at least 9 yrs of schooling | -0.005 |
|  | (0.034) |
| Primary caregiver has non-farm work | -0.036 |
|  | (0.048) |
| Household asset index | -0.006 |
|  | (0.016) |
| Father out-migrated | 0.020 |
|  | (0.026) |
| Constant | 0.320*** |
|  | (0.065) |
| Observations | 814 |
| R-square | 0.26 |
| Overall Attrition Rate | 0.167 |
| In the regression, we control for village fixed effects. Standard errors in the parentheses are clustered at the village level. * p < 0.05, ** p < 0.01, *** p < 0.001." | |
