# Supplementary material for: Correlates of participation in community-based interventions: Evidence from a parenting program in rural China
Source: PLoS One. 2020 Sep 8;15(9):e0238841. doi: 10.1371/journal.pone.0238841 (PMC7478867; doi:10.1371/journal.pone.0238841)
Supplement: S2 Table — (DOCX) [file pone.0238841.s008.docx]

**S2 Table. Distribution of social ties with two alternative social tie definitions.**

| Panel A: Distribution of number of social ties (defined as interacting at least once per month) | | | |
| --- | --- | --- | --- |
|  | | Percentage |  |
|  | 0-2 friends | 17.30% |  |
|  | 3-4 friends | 22.58% |  |
|  | 5-6 friends | 24.78% |  |
|  | ≥ 7 friends | 35.34% |  |
| Panel B: Distribution of number of social ties (defined as interacting at least twice per week) | | |  |
|  | | Percentage |  |
|  | 0-2 friends | 36.80% |  |
|  | 3-4 friends | 29.18% |  |
|  | 5-6 friends | 17.45% |  |
|  | ≥ 7 friends | 16.57% |  |
| Source: Authors' survey | | |  |
