# Supplementary material for: Correlates of participation in community-based interventions: Evidence from a parenting program in rural China
Source: PLoS One. 2020 Sep 8;15(9):e0238841. doi: 10.1371/journal.pone.0238841 (PMC7478867; doi:10.1371/journal.pone.0238841)
Supplement: S3 Table — (DOCX) [file pone.0238841.s009.docx]

**S3 Table. Correlates of participation in the community-based ECD program** **(social ties defined as interacting at least once a month).**

|  | (1) | (2) | (3) | (4) | |
| --- | --- | --- | --- | --- | --- |
|  | Participation rate | | | | |
| Number of social ties |  | 0.026*** | 0.026*** | 0.026*** | |
|  |  | (0.005) | (0.005) | (0.005) | |
| Average distance of social ties to program |  |  | -0.008 | -0.002 | |
|  |  |  | (0.016) | (0.012) | |
| Average participation of social ties |  |  |  | 0.142 | |
|  |  |  |  | (0.136) | |
| Distance to the program (km) | -0.091*** | -0.074** | -0.072** | -0.070** | |
|  | (0.022) | (0.024) | (0.025) | (0.025) | |
| Male child | 0.009 | 0.008 | 0.007 | 0.006 | |
|  | (0.020) | (0.020) | (0.020) | (0.020) | |
| Child age (month) | 0.002 | 0.002 | 0.002 | 0.002 | |
|  | (0.002) | (0.002) | (0.002) | (0.002) | |
| Standardized BSID-III Cognitive Score | 0.014 | 0.013 | 0.013 | 0.012 | |
|  | (0.010) | (0.010) | (0.010) | (0.010) | |
| Only child | -0.062* | -0.047 | -0.048 | -0.048 | |
|  | (0.024) | (0.025) | (0.025) | (0.025) | |
| Grandparent is primary caregiver | 0.048 | 0.036 | 0.036 | 0.035 | |
|  | (0.025) | (0.025) | (0.025) | (0.025) | |
| Primary caregiver has at least 9 yrs of schooling | -0.006 | -0.004 | -0.004 | -0.004 | |
|  | (0.031) | (0.031) | (0.032) | (0.032) | |
| Primary caregiver has non-farm work | -0.005 | 0.012 | 0.013 | 0.007 | |
|  | (0.036) | (0.039) | (0.039) | (0.038) | |
| Household asset index | 0.004 | -0.002 | -0.002 | -0.000 | |
|  | (0.015) | (0.013) | (0.013) | (0.012) | |
| Father out-migrated | -0.002 | -0.009 | -0.009 | -0.010 | |
|  | (0.017) | (0.016) | (0.016) | (0.016) | |
| Constant | 0.342*** | 0.245*** | 0.247*** | 0.200* | |
|  | (0.040) | (0.049) | (0.048) | (0.076) | |
| Observations | 670 | 670 | 670 | 670 | |
| R-squared | 0.37 | 0.42 | 0.42 | 0.43 | |
| In the regression, we control for village fixed effects. Standard errors in the parentheses are clustered at the village level. | | | | |  |
| * p < 0.05, ** p < 0.01, *** p < 0.001." | | | | |  |
