# Supplementary material for: Correlates of participation in community-based interventions: Evidence from a parenting program in rural China
Source: PLoS One. 2020 Sep 8;15(9):e0238841. doi: 10.1371/journal.pone.0238841 (PMC7478867; doi:10.1371/journal.pone.0238841)
Supplement: S4 Table — (DOCX) [file pone.0238841.s010.docx]

**S4 Table. Correlates of participation in the community-based ECD program (social ties defined as interacting at least twice a week).**

|  | (1) | (2) | (3) | (4) | |  |
| --- | --- | --- | --- | --- | --- | --- |
|  | Participation rate | | | | | |
| Number of social ties |  | 0.026*** | 0.026*** | 0.024*** | |  |
|  |  | (0.005) | (0.006) | (0.006) | |  |
| Average distance of social ties to program |  |  | -0.004 | -0.002 | |  |
|  |  |  | (0.018) | (0.017) | |  |
| Average participation of social ties |  |  |  | 0.186* | |  |
|  |  |  |  | (0.088) | |  |
| Distance to the program (km) | -0.091*** | -0.076** | -0.075** | -0.069** | |  |
|  | (0.022) | (0.023) | (0.025) | (0.024) | |  |
| Male child | 0.009 | 0.010 | 0.010 | 0.007 | |  |
|  | (0.020) | (0.020) | (0.020) | (0.019) | |  |
| Child age (month) | 0.002 | 0.001 | 0.001 | 0.001 | |  |
|  | (0.002) | (0.002) | (0.002) | (0.002) | |  |
| Standardized BSID-III Cognitive Score | 0.014 | 0.013 | 0.013 | 0.013 | |  |
|  | (0.010) | (0.010) | (0.010) | (0.010) | |  |
| Only child | -0.062* | -0.049 | -0.050 | -0.051* | |  |
|  | (0.024) | (0.025) | (0.025) | (0.024) | |  |
| Grandparent is primary caregiver | 0.048 | 0.040 | 0.041 | 0.043 | |  |
|  | (0.025) | (0.026) | (0.026) | (0.026) | |  |
| Primary caregiver has at least 9 yrs of schooling | -0.006 | -0.002 | -0.002 | -0.001 | |  |
|  | (0.031) | (0.030) | (0.030) | (0.030) | |  |
| Primary caregiver has non-farm work | -0.005 | 0.008 | 0.007 | 0.003 | |  |
|  | (0.036) | (0.038) | (0.038) | (0.038) | |  |
| Household asset index | 0.004 | -0.003 | -0.003 | 0.000 | |  |
|  | (0.015) | (0.014) | (0.013) | (0.012) | |  |
| Father out-migrated | -0.002 | -0.013 | -0.013 | -0.013 | |  |
|  | (0.017) | (0.018) | (0.017) | (0.016) | |  |
| Constant | 0.342*** | 0.289*** | 0.290*** | 0.244*** | |  |
|  | (0.040) | (0.044) | (0.043) | (0.051) | |  |
| Observations | 670 | 670 | 670 | 670 | |  |
| R-squared | 0.37 | 0.42 | 0.42 | 0.43 | |  |
| In the regression, we control for village fixed effects. Standard errors in the parentheses are clustered at the village level. | | | | |  |  |
| * p < 0.05, ** p < 0.01, *** p < 0.001." | | | | |  |  |
