# Supplementary material for: Correlates of participation in community-based interventions: Evidence from a parenting program in rural China
Source: PLoS One. 2020 Sep 8;15(9):e0238841. doi: 10.1371/journal.pone.0238841 (PMC7478867; doi:10.1371/journal.pone.0238841)
Supplement: S5 Table — (DOCX) [file pone.0238841.s011.docx]

**S5 Table. Correlates of participation in the community-based ECD program (adding distance*grandparents as main caregiver).**

|  | (1) | (2) | (3) | (4) | |  |
| --- | --- | --- | --- | --- | --- | --- |
|  | Participation rate | | | | | |
| Number of social ties |  | 0.025*** | 0.025*** | 0.024*** | |  |
|  |  | (0.006) | (0.006) | (0.006) | |  |
| Average distance of social ties to program |  |  | 0.003 | 0.009 | |  |
|  |  |  | (0.014) | (0.011) | |  |
| Average participation of social ties |  |  |  | 0.212* | |  |
|  |  |  |  | (0.098) | |  |
| Distance to the program (km) | -0.081*** | -0.065** | -0.066** | -0.062* | |  |
|  | (0.021) | (0.023) | (0.025) | (0.024) | |  |
| Distance to the program (km)* Grandparent as main caregiver | -0.085** | -0.080** | -0.081** | -0.078** | |  |
|  | (0.031) | (0.028) | (0.028) | (0.026) | |  |
| Male Child | 0.012 | 0.012 | 0.012 | 0.009 | |  |
|  | (0.020) | (0.020) | (0.019) | (0.019) | |  |
| Age of child (months) | 0.002 | 0.002 | 0.002 | 0.002 | |  |
|  | (0.002) | (0.002) | (0.002) | (0.002) | |  |
| Standardized BSID-III cognitive score | 0.011 | 0.010 | 0.010 | 0.009 | |  |
|  | (0.010) | (0.010) | (0.010) | (0.010) | |  |
| Only child | -0.057* | -0.043 | -0.043 | -0.044 | |  |
|  | (0.025) | (0.025) | (0.025) | (0.024) | |  |
| Grandparent is main caregiver | 0.117*** | 0.100** | 0.101** | 0.097** | |  |
|  | (0.033) | (0.033) | (0.033) | (0.031) | |  |
| Primary caregiver has at least 9 yrs of schooling | -0.007 | -0.008 | -0.008 | -0.011 | |  |
|  | (0.030) | (0.030) | (0.031) | (0.030) | |  |
| Primary caregiver has non-farm work | -0.003 | 0.009 | 0.009 | -0.003 | |  |
|  | (0.036) | (0.039) | (0.039) | (0.038) | |  |
| Household asset index | 0.002 | -0.003 | -0.003 | 0.002 | |  |
|  | (0.015) | (0.013) | (0.013) | (0.012) | |  |
| Father out-migrated | -0.008 | -0.014 | -0.014 | -0.013 | |  |
|  | (0.017) | (0.017) | (0.017) | (0.016) | |  |
| Constant | 0.321*** | 0.247*** | 0.246*** | 0.185** | |  |
|  | (0.042) | (0.049) | (0.048) | (0.061) | |  |
| Observations | 670 | 670 | 670 | 670 | |  |
| R-squared | 0.37 | 0.42 | 0.42 | 0.43 | |  |
| In the regression, we control for village fixed effects. Standard errors in the parentheses are clustered at the village level. | | | | |  |  |
| * p < 0.05, ** p < 0.01, *** p < 0.001." | | | | |  |  |
