# Supplementary material for: Correlates of participation in community-based interventions: Evidence from a parenting program in rural China
Source: PLoS One. 2020 Sep 8;15(9):e0238841. doi: 10.1371/journal.pone.0238841 (PMC7478867; doi:10.1371/journal.pone.0238841)
Supplement: Attachment — Submitted filename: Response to Reviewers.docx [file pone.0238841.s012.docx]

PONE-D-19-28402

Title: Correlates of participation in community-based interventions: evidence from a parenting program in rural China

Journal: PLOS ONE

## Dear Professor Mughal:

We would like to thank you and the referees for comments on our manuscript and for the opportunity to submit our revisions. We have addressed four issues that you raised about our submission and additional questions about our data policy. Specifically, in the re-submission, we have:

- Reformatted the manuscript according to the journal’s requirement;
- Uploaded the survey questionnaires both in Chinese and in English;
- Corrected an error in our list of funding sources and amended our competing interest declaration in the cover letter;
- Explained the reason why the data are only available upon request in the cover letter.

Additionally, we believe that the referees’ comments have been helpful, and we have incorporated all of their suggestions into the revised manuscript. Specifically, we have:

- Revised the order of our literature review, results and discussion to present the correlations-to-social ties before correlations-to-distance;
- Added a statistical analysis exploring the interactions between grandparent caregivers and geographic distance to support one of the hypotheses raised in the manuscript;
- Corrected a typo in our definition of social ties, and added a sensitivity analysis of the definition of social ties by comparing the results of a stricter versus looser definition of social ties among respondents in the sample.

All of these revisions have been made with tracked changes in the manuscript. In the following pages, we have included detailed, comment-by-comment responses to you and each reviewer to facilitate your re-review.

In making this resubmission, we would like to reiterate that we have not submitted this manuscript to any other journal while it is under review by *PLOS ONE*.

Thank you for your consideration. We look forward to hearing from you.

Sincerely,

Yiwei Qian

University of Southern California

Los Angeles, CA, USA

**Response to the Editor**

**Comment (1)**

Please amend your list of authors on the manuscript to ensure that each author is linked to an affiliation.

**Response to comment (1)**

We have amended the list of authors on the manuscript so that each author is linked to an affiliation.

**Comment (2)**

You have indicated that data underlying your study cannot be shared publicly. Before we proceed with your manuscript, please address the following points:

**a)** PLOS requires all data be made publicly available, barring specific ethical and legal restrictions. Please see [https://urldefense.com/v3/__https://journals.plos.org/plosone/s/data-availability*loc-acceptable-data-access-restrictions__;Iw!!LIr3w8kk_Xxm!885EBwlfT21Dg8V1_SWOYbTrd9g0acl4PAWQ7ND3Da_au0ShKWjVAADBCpSPM0BJ$](https://urldefense.com/v3/__https:/journals.plos.org/plosone/s/data-availability*loc-acceptable-data-access-restrictions__;Iw!!LIr3w8kk_Xxm!885EBwlfT21Dg8V1_SWOYbTrd9g0acl4PAWQ7ND3Da_au0ShKWjVAADBCpSPM0BJ$)  and elaborate as to which specific restrictions have been imposed on data sharing.

**b)** Please name the collaborators that have imposed the ethical restrictions on data sharing.

**c)** You have listed a co-author as the sole point of contact for data requests. Please provide a third-party, non-author point of contact to which data access requests can be sent (such as your institution's data access committee).

**Response to comment (2)**

Thank you for raising these points. We have responded to each point separately below.

**a)** Our data cannot be shared publicly because the data contains sensitive data on the cognitive development of infants in rural China.

**b)** The university authorities of our collaborator—Yaojiang Shi, Center for Experimental Economics of Education, Shaanxi Normal University—have requested that the data used in this study not be publicly shared in order to protect the confidential information on the cognitive development of infants in our study.

**c)** We have revised the point of contact for data requests to Sean Sylvia, School of Public Health, University of North Carolina (contact via [sean_sylvia@unc.edu](mailto:sean_sylvia@unc.edu)). Sean Sylvia is not a coauthor of the paper.

Above all, we would like to revise our data statement to the following:

“Data cannot be shared publicly because the data contains sensitive information on the cognitive development of infants in rural China. Data from the study are available upon request from Sean Sylvia, School of Public Health, University of North Carolina (contact via sean_sylvia@unc.edu) for researchers who meet the criteria for access to confidential data.”

**Response to the Editor**

**Editor Comment 1.**

Please ensure that your manuscript meets PLOS ONE's style requirements, including those for file naming. The PLOS ONE style templates can be found at

<http://www.journals.plos.org/plosone/s/file?id=wjVg/PLOSOne_formatting_sample_main_body.pdf> and <http://www.journals.plos.org/plosone/s/file?id=ba62/PLOSOne_formatting_sample_title_authors_affiliations.pdf>

**Response to Editor Comment 1:**

We have reformatted our manuscript to meet the journal’s style requirement.

**Editor Comment 2.**

Please include additional information regarding the survey or questionnaire used in the study and ensure that you have provided sufficient details that others could replicate the analyses. For instance, if you developed a questionnaire as part of this study and it is not under a copyright more restrictive than CC-BY, please include a copy, in both the original language and English, as Supporting Information.

**Response to Editor Comment 2:**

We uploaded the survey questionnaires used in the data collection, both in Chinese and in English, in the re-submission as supporting information.

**Editor Comment 3.**

Thank you for stating the following in the Financial Disclosure section:

"SR received funding from the 111 Project (Grant no: B16031). The 111 project is funded by the Ministry of Education, China. The url to the funder information (http://www.moe.gov.cn/srcsite/A16/s7062/200608/t20060830_82287.html) is only in Chinese.

SR received funding from the National Natural Science Foundation of China (Grant no. 71703083). The url to the funder information can be found at <http://www.nsfc.gov.cn/english/site_1/index.html>;

SR received funding from the International Initiative for Impact Evaluation (3ie). The grant number in this case is not applicable. The url to the funder information can be found at <https://www.3ieimpact.org>;

SR received funding from the UBS Optimus Foundation. The grant number in this case is not applicable. The url to the funder information can be found at https://www.ubs.com/microsites/optimus-foundation/en.html;

SR received funding from the China Medical Board. The grant number in this case is not applicable. The url to the funder information can be found at https://chinamedicalboard.org;

SR received funding from the Bank of East Asia. The grant number in this case is not applicable. The url to the funder information can be found at https://www.hkbea.com/html/en/index.html;

SR received funding from the HBGDki Initiative. The HBGDki Initiative is funded by the Bill and Melinda Gates Foundation. The grant number in this case is not applicable. The url to the funder information can be found at https://www.gatesfoundation.org;

The funders had no role in study design, data collection and analysis, decision to publish, or preparation of the manuscript. "

Thank you for stating the following in the Competing Interests section:"The authors have declared that no competing interests exist."

We note that you received funding from a commercial source: "Bank of East Asia"

Please provide an amended Competing Interests Statement that explicitly states this commercial funder, along with any other relevant declarations relating to employment, consultancy, patents, products in development, marketed products, etc.

Within this Competing Interests Statement, please confirm that this does not alter your adherence to all PLOS ONE policies on sharing data and materials by including the following statement: "This does not alter our adherence to PLOS ONE policies on sharing data and materials.” (as detailed online in our guide for authors http://journals.plos.org/plosone/s/competing-interests). If there are restrictions on sharing of data and/or materials, please state these. Please note that we cannot proceed with consideration of your article until this information has been declared.

Please include your amended Competing Interests Statement within your cover letter. We will change the online submission form on your behalf.

**Response to Editor Comment 3:**

Thank you for noting the list of funding sources included in this paper and pointing out the Bank of East Asia in this list. After examining the funding list, we have realized that we erroneously included funders who had supported a separate study unrelated to this manuscript. The National Natural Science Foundation of China, UBS Optimus Foundation, China Medical Board, and the HBGDki Initiative provided funding to support a study of another early childhood development intervention program conducted in a similar area of rural China; however, they did not provide financial support for the early childhood development program or the study reported in this manuscript. We apologize for this confusion.

In response to the Editor’s comment, we have carefully reviewed the list of funding sources for the present study and removed sources of funding that did not support the present study. In the revised cover, we have added the following paragraph as our amended Completing Interests statement:

“The funding sources that supported the study are the International Initiative for Impact Evaluation (3ie) and the 111 Project (Grant no: B16031). Neither of the funding sources for the present source are commercial sources. None of the funders had any role in study design, data collection and analysis, decision to publish, or preparation of the manuscript.”

In addition, this does not alter our adherence to PLOS ONE policies on sharing data and materials

**Editor Comment 4.**

We note that you have indicated that data from this study are available upon request. PLOS only allows data to be available upon request if there are legal or ethical restrictions on sharing data publicly. For information on unacceptable data access restrictions, please see <http://journals.plos.org/plosone/s/data-availability#loc-unacceptable-data-access-restrictions>.

In your revised cover letter, please address the following prompts:

a) If there are ethical or legal restrictions on sharing a de-identified data set, please explain them in detail (e.g., data contain potentially identifying or sensitive patient information) and who has imposed them (e.g., an ethics committee). Please also provide contact information for a data access committee, ethics committee, or other institutional body to which data requests may be sent.

b) If there are no restrictions, please upload the minimal anonymized data set necessary to replicate your study findings as either Supporting Information files or to a stable, public repository and provide us with the relevant URLs, DOIs, or accession numbers. Please see <http://www.bmj.com/content/340/bmj.c181.long>for guidelines on how to de-identify and prepare clinical data for publication. For a list of acceptable repositories, please see <http://journals.plos.org/plosone/s/data-availability#loc-recommended-repositories>.

We will update your Data Availability statement on your behalf to reflect the information you provide.

**Response to Editor Comment 4:**

Since we received additional questions about our data policy, we have revised our response to this comment.

**New response:**

Our data cannot be shared publicly because the data contains sensitive data on the cognitive development of infants in rural China. The university authorities of our collaborator—Yaojiang Shi, Center for Experimental Economics of Education, Shaanxi Normal University—have requested that the data used in this study not be publicly shared in order to protect the confidential information on the cognitive development of infants in our study. We have revised the point of contact for data requests to Sean Sylvia, School of Public Health, University of North Carolina (contact via [sean_sylvia@unc.edu](mailto:sean_sylvia@unc.edu)). Sean Sylvia is not a coauthor of the paper.

Above all, we would like to revise our data statement to the following:

“Data cannot be shared publicly because the data contains sensitive information on the cognitive development of infants in rural China. Data from the study are available upon request from Sean Sylvia, School of Public Health, University of North Carolina (contact via sean_sylvia@unc.edu) for researchers who meet the criteria for access to confidential data.”

**Previous response:**

Because of privacy restrictions imposed on us by our collaborators in China, we cannot upload the data to an online repository. However, we are happy to share the data with any researchers who contact us directly.

In response to the Editor’s comment, in the revised cover letter, we have added the following paragraph as our Data Availability statement:

“Due to privacy restrictions imposed on the research team by our collaborators in China, we cannot upload the data to an online repository. Data from the study are available from the Rural Education Action Program, Freeman Spogli Institute for International Studies, Stanford University (contact via rozelle@stanford.edu) for researchers who meet the criteria for access to confidential data.”

**Response to Reviewer 1**

**General Comment 1:** The authors examine which characteristics predict participation in early childhood development programs in rural China. Proximity, social ties, peer participation are each significant predictors.

**Response to General Comment 1:** Thank you for your thoughtful read.

**Comment 1:** Providing parenting centers throughout rural China is a very interesting intervention and participation is an important component of understanding the overall effects and costs/benefits. The emphasis throughout the paper seems to be the effects of distance first, followed by social ties. I would recommend (though not require) reversing this ordering of emphasis. Focusing on the association between participation and distance is less novel because the relationship is self-evident.

**Response to Comment 1.** We thank the reviewer for this suggestion. In the revised manuscript, we have reversed the emphasis to describe the role of social ties first. In doing so, we have made structural changes throughout the manuscript. All of these revisions were made with tracked changes in the manuscript for your review.

Additionally, we would like to note that we believe both distance and social ties are equally important correlates of participation to study in the context of a community-based early childhood development (ECD) program. To date, there is a general lack of quantitative evidence on the correlates of participation in community-based ECD programs. As such, our findings of both distance and social ties offer a contribution to the literature, particularly in a developing setting such as rural China, where there is a need for increased ECD investment.

**Comment 2:** The authors make in interesting point in the discussion regarding the role of distance and the mobility of the caretakers: that geographic barriers are not due to distance alone but issues of low mobility. However, the authors do not provide evidence of this. Please add distance*grandparent caretaker to the models of participation in order to support the claim. If supported, this would add to the interest of the geographic point and perhaps justify its emphasis over the social ties.

**Response to Comment 2.** Thank you for your thoughtful suggestion.

We have re-performed our correlational analysis with an additional interaction term between distance to the parenting center and grandparents as the primary caregiver. The results are presented in S5 Table (included at the end of this letter to facilitate your review). In our most preferred specification (S5 Table, column 4), we find that distance serves as much greater barrier for participation among grandparents compared. For all households, one kilometer corresponds to a 6.2-percentage-point decrease in participation (row 4). For households in which a grandparent is the child’s primary caregiver, however, a one-kilometer increase in distance corresponds to an additional decrease in participation by 7.8 percentage points (row 5), summing to a 15-percentage-point decrease in participation in total (row 4 plus row 5). This result supports our point that the geographic barriers are not due to distance alone, but also to the low mobility of elderly caregivers.

In the revised manuscript, we have added a discussion of S5 Table on Page 23:

“To empirically examine whether the low mobility of grandparent caregivers is contributing to the negative correlation between geographic proximity and program participation in our study, we conducted the same multivariate regression as Table 5 with an additional interaction term between “distance to the parenting center” and “grandparent as the primary caregiver.” The results are presented in S5 Table. In our most preferred specification (S5 Table, column 4), we find that distance serves as a much greater barrier for participation among grandparents. For all households, a one-kilometer increase in distance corresponds to a 6.2-percentage-point decrease in participation (row 4). For all households, one kilometer corresponds to a 6.2-percentage-point decrease in participation (row 4). For households in which a grandparent is the child’s primary caregiver, however, a one-kilometer increase in distance corresponds to an additional decrease in participation by 7.8 percentage points (row 5), summing to a 15-percentage-point decrease in total (row 4 plus row 5). Therefore, for community-based ECD programs in rural China, geographic barriers are not only due to distance, but also to issues of low mobility among elderly caregivers, who make up a relatively large share of the primary caregivers in our sample. Considering these barriers, policies or programs that could lessen the physical burden of travel would likely have a significant positive effect on participation.”

We also changed the covariate “mother is primary caregiver” to “grandparent is primary caregiver” in Table 5 and S1 Table for consistency in the analysis. The updated results in Table 5 and Table S1 are almost the same as that in Table 5 and S1 Table in the original manuscript.

**Comment 3:** How was it determined that 3+ interactions = social tie? 3 seems like a high bar and households that interact 1 or 2 times per week would also seem to be socially tied. Sensitivity analyses should be performed around this threshold.

**Response to Comment 3.** Thank you for this note. We agree with the reviewer that households interacting 1 or 2 times per week might also be deemed “socially tied.” In fact, would like to apologize for a typo in the manuscript regarding our definition of a social tie. In our analysis, households that interacted at least once per week (rather than three times per week) are considered to have a social tie. We have corrected this typo in the revised manuscript, and we have done a careful check for any other typos.

In addition, we conducted a sensitivity analysis of the definition of social ties. In S2 Table, we first show the distribution of the number of social ties, with social ties defined as interacting at least once per month (less frequent than the original definition) and interacting at least twice per week (more frequent than the original definition), respectively. Next, in S3 Table and S4 Table, we present the correlates of parenting center participation rates. The results find consistent correlations between the number of social ties and participation in the parenting center, regardless of the definition for a social tie. Thus, in S3 Table and S5 Table, as in Table 5 of the original manuscript, the number of social ties is consistently and positively correlated with participation.

When we examine the correlation between the average participation of social ties and individual participation, we find that when we define social ties as interacting at least twice per week (S4 Table), the correlations are significant and positive, consistent with our original Table 5. However, the correlation is not statistically significant when we use a looser definition of social ties (at least once a month; S3 Table), although the coefficient is still positive. These results demonstrate that our original definition of social ties (interacting at least once per week) is an adequate measure.

In the revised manuscript, we have added a short discussion of S2, S3 and S4 Tables to confirm the validity of our definition of social ties (page 19):

“To check the robustness of our findings, we performed the same multivariate regression analysis as Table 5, using two different definitions social ties. S2 Table shows the distribution of number of social ties in our sample, using two alternative definitions for social ties. Panel A shows the distribution when social ties are defined as interacting at least once per month (less frequent than the original definition), and Panel B presents the distribution when social ties are defined as interacting at least twice per week (more frequent than the original definition). S3 Table and S4 Table present the regression results with social ties defined as interacting at least once per month and defined as interacting at least twice per week, respectively. The results find consistent correlations between the number of social ties and program participation, regardless of the definition of a social tie. However, although correlation between the average participation rate of social ties and individual participation remains significant when we use the stricter definition of a social tie (at least twice a week; S4 Table), the correlation is not statistically significant when we use the looser definition of social ties (at least once a month; S3 Table). Geographic proximity remains significantly correlated to program participation at a similar magnitude in all tables.”

**S2 Table. Distribution of social ties with two alternative definitions of social ties**

| Panel A: Distribution of number of social ties (defined as interacting at least once per month) | | | |
| --- | --- | --- | --- |
|  | | Percentage |  |
|  | 0-2 friends | 17.30% |  |
|  | 3-4 friends | 22.58% |  |
|  | 5-6 friends | 24.78% |  |
|  | ≥ 7 friends | 35.34% |  |
| Panel B: Distribution of number of social ties (defined as interacting at least twice per week) | | |  |
|  | | Percentage |  |
|  | 0-2 friends | 36.80% |  |
|  | 3-4 friends | 29.18% |  |
|  | 5-6 friends | 17.45% |  |
|  | ≥ 7 friends | 16.57% |  |
| Source: Authors' survey | | |  |

**S3 Table. Correlates of participation in the community-based ECD program** **(social ties defined as interacting at least once a month)**

|  | (1) | (2) | (3) | (4) | |
| --- | --- | --- | --- | --- | --- |
|  | Participation rate | | | | |
| Number of social ties |  | 0.026*** | 0.026*** | 0.026*** | |
|  |  | (0.005) | (0.005) | (0.005) | |
| Average distance of social ties to program |  |  | -0.008 | -0.002 | |
|  |  |  | (0.016) | (0.012) | |
| Average participation of social ties |  |  |  | 0.142 | |
|  |  |  |  | (0.136) | |
| Distance to the program (km) | -0.091*** | -0.074** | -0.072** | -0.070** | |
|  | (0.022) | (0.024) | (0.025) | (0.025) | |
| Male Child | 0.009 | 0.008 | 0.007 | 0.006 | |
|  | (0.020) | (0.020) | (0.020) | (0.020) | |
| Age of child (months) | 0.002 | 0.002 | 0.002 | 0.002 | |
|  | (0.002) | (0.002) | (0.002) | (0.002) | |
| Standardized BSID-III cognitive score | 0.014 | 0.013 | 0.013 | 0.012 | |
|  | (0.010) | (0.010) | (0.010) | (0.010) | |
| Only child | -0.062* | -0.047 | -0.048 | -0.048 | |
|  | (0.024) | (0.025) | (0.025) | (0.025) | |
| Grandparent is primary caregiver | 0.048 | 0.036 | 0.036 | 0.035 | |
|  | (0.025) | (0.025) | (0.025) | (0.025) | |
| Primary caregiver has at least 9 yrs of schooling | -0.006 | -0.004 | -0.004 | -0.004 | |
|  | (0.031) | (0.031) | (0.032) | (0.032) | |
| Primary caregiver has non-farm work | -0.005 | 0.012 | 0.013 | 0.007 | |
|  | (0.036) | (0.039) | (0.039) | (0.038) | |
| Asset index | 0.004 | -0.002 | -0.002 | -0.000 | |
|  | (0.015) | (0.013) | (0.013) | (0.012) | |
| Father out-migrated | -0.002 | -0.009 | -0.009 | -0.010 | |
|  | (0.017) | (0.016) | (0.016) | (0.016) | |
| Constant | 0.342*** | 0.245*** | 0.247*** | 0.200* | |
|  | (0.040) | (0.049) | (0.048) | (0.076) | |
| Observations | 670 | 670 | 670 | 670 | |
| R-squared | 0.37 | 0.42 | 0.42 | 0.43 | |
| In the regression, we control for village fixed effects. Standard errors in the parentheses are clustered at the village level. | | | | |  |
| * p < 0.05, ** p < 0.01, *** p < 0.001." | | | | |  |

**S4 Table. Correlates of participation in the community-based ECD program (social ties defined as interacting at least twice a week)**

|  | (1) | (2) | (3) | (4) | |  |
| --- | --- | --- | --- | --- | --- | --- |
|  | Participation rate | | | | | |
| Number of social ties |  | 0.026*** | 0.026*** | 0.024*** | |  |
|  |  | (0.005) | (0.006) | (0.006) | |  |
| Average distance of social ties to program |  |  | -0.004 | -0.002 | |  |
|  |  |  | (0.018) | (0.017) | |  |
| Average participation of social ties |  |  |  | 0.186* | |  |
|  |  |  |  | (0.088) | |  |
| Distance to the program (km) | -0.091*** | -0.076** | -0.075** | -0.069** | |  |
|  | (0.022) | (0.023) | (0.025) | (0.024) | |  |
| Male Child | 0.009 | 0.010 | 0.010 | 0.007 | |  |
|  | (0.020) | (0.020) | (0.020) | (0.019) | |  |
| Age of child (months) | 0.002 | 0.001 | 0.001 | 0.001 | |  |
|  | (0.002) | (0.002) | (0.002) | (0.002) | |  |
| Standardized BSID-III cognitive score | 0.014 | 0.013 | 0.013 | 0.013 | |  |
|  | (0.010) | (0.010) | (0.010) | (0.010) | |  |
| Only child | -0.062* | -0.049 | -0.050 | -0.051* | |  |
|  | (0.024) | (0.025) | (0.025) | (0.024) | |  |
| Grandparent is primary caregiver | 0.048 | 0.040 | 0.041 | 0.043 | |  |
|  | (0.025) | (0.026) | (0.026) | (0.026) | |  |
| Primary caregiver has at least 9 yrs of schooling | -0.006 | -0.002 | -0.002 | -0.001 | |  |
|  | (0.031) | (0.030) | (0.030) | (0.030) | |  |
| Primary caregiver has non-farm work | -0.005 | 0.008 | 0.007 | 0.003 | |  |
|  | (0.036) | (0.038) | (0.038) | (0.038) | |  |
| Asset index | 0.004 | -0.003 | -0.003 | 0.000 | |  |
|  | (0.015) | (0.014) | (0.013) | (0.012) | |  |
| Father out-migrated | -0.002 | -0.013 | -0.013 | -0.013 | |  |
|  | (0.017) | (0.018) | (0.017) | (0.016) | |  |
| Constant | 0.342*** | 0.289*** | 0.290*** | 0.244*** | |  |
|  | (0.040) | (0.044) | (0.043) | (0.051) | |  |
| Observations | 670 | 670 | 670 | 670 | |  |
| R-squared | 0.37 | 0.42 | 0.42 | 0.43 | |  |
| In the regression, we control for village fixed effects. Standard errors in the parentheses are clustered at the village level. | | | | |  |  |
| * p < 0.05, ** p < 0.01, *** p < 0.001." | | | | |  |  |

**S5 Table. Correlates of participation in the community-based ECD program (adding distance*grandparents as primary caregiver)**

|  | (1) | (2) | (3) | (4) | |  |
| --- | --- | --- | --- | --- | --- | --- |
|  | Participation rate | | | | | |
| Number of social ties |  | 0.025*** | 0.025*** | 0.024*** | |  |
|  |  | (0.006) | (0.006) | (0.006) | |  |
| Average distance of social ties to program |  |  | 0.003 | 0.009 | |  |
|  |  |  | (0.014) | (0.011) | |  |
| Average participation of social ties |  |  |  | 0.212* | |  |
|  |  |  |  | (0.098) | |  |
| Distance to the program (km) | -0.081*** | -0.065** | -0.066** | -0.062* | |  |
|  | (0.021) | (0.023) | (0.025) | (0.024) | |  |
| Distance to the program (km)* Grandparent is primary caregiver | -0.085** | -0.080** | -0.081** | -0.078** | |  |
|  | (0.031) | (0.028) | (0.028) | (0.026) | |  |
| Male Child | 0.012 | 0.012 | 0.012 | 0.009 | |  |
|  | (0.020) | (0.020) | (0.019) | (0.019) | |  |
| Age of child (months) | 0.002 | 0.002 | 0.002 | 0.002 | |  |
|  | (0.002) | (0.002) | (0.002) | (0.002) | |  |
| Standardized BSID-III cognitive score | 0.011 | 0.010 | 0.010 | 0.009 | |  |
|  | (0.010) | (0.010) | (0.010) | (0.010) | |  |
| Only child | -0.057* | -0.043 | -0.043 | -0.044 | |  |
|  | (0.025) | (0.025) | (0.025) | (0.024) | |  |
| Grandparent is primary caregiver | 0.117*** | 0.100** | 0.101** | 0.097** | |  |
|  | (0.033) | (0.033) | (0.033) | (0.031) | |  |
| Primary caregiver has at least 9 yrs of schooling | -0.007 | -0.008 | -0.008 | -0.011 | |  |
|  | (0.030) | (0.030) | (0.031) | (0.030) | |  |
| Primary caregiver has non-farm work | -0.003 | 0.009 | 0.009 | -0.003 | |  |
|  | (0.036) | (0.039) | (0.039) | (0.038) | |  |
| Asset index | 0.002 | -0.003 | -0.003 | 0.002 | |  |
|  | (0.015) | (0.013) | (0.013) | (0.012) | |  |
| Father out-migrated | -0.008 | -0.014 | -0.014 | -0.013 | |  |
|  | (0.017) | (0.017) | (0.017) | (0.016) | |  |
| Constant | 0.321*** | 0.247*** | 0.246*** | 0.185** | |  |
|  | (0.042) | (0.049) | (0.048) | (0.061) | |  |
| Observations | 670 | 670 | 670 | 670 | |  |
| R-squared | 0.37 | 0.42 | 0.42 | 0.43 | |  |
| In the regression, we control for village fixed effects. Standard errors in the parentheses are clustered at the village level. | | | | |  |  |
| * p < 0.05, ** p < 0.01, *** p < 0.001." | | | | |  |  |

**Response to Reviewer 2**

**General Comment 1:** The manuscript collected data of all children in the desired age range and their caregivers in randomly selected 100 village from 20 nationally-designated poverty counties. The statistical analysis been performed appropriately and rigorously，the conclusions has been drawn appropriately based on the data presented and statistical analysis.
The data are only available with some restrictions because of the involvement of human participants, data are available for researchers who meet the criteria for access to confidential data.
The manuscript presented in an intelligible fashion and written in very good English.
The research is valuable because this paper is one of the first to quantitatively document program participation in a community-based ECD intervention, and is the first paper to examine factors that may predict participation in community-based ECD interventions.

**Response to General Comment 1:** Thank you for your thoughtful read of this paper and your kind words.
